# Reported Methods, Distributions, and Frequencies of Torture Globally: A Systematic Review and Meta-Analysis

**DOI:** 10.1001/jamanetworkopen.2023.36629

**Published:** 2023-10-03

**Authors:** Andrew Milewski, Eliana Weinstein, Jacob Lurie, Annabel Lee, Faten Taki, Tara Pilato, Caroline Jedlicka, Gunisha Kaur

**Affiliations:** 1 Department of Anesthesiology, NewYork-Presbyterian Hospital/Weill Cornell Medicine, New York; 2 Weill Cornell Medicine Medical College, New York, New York; 3 Samuel J. Wood Library, Weill Cornell Medicine, New York, New York; 4 Kingsborough Community College, City University of New York, Brooklyn

## Abstract

**Importance:**

Despite its prohibition by the United Nations Convention against Torture and other international treaties, torture has been perpetrated against countless individuals worldwide, and health care practitioners globally are increasingly encountering refugee torture survivors in their clinical practices. The methods, geographic distribution, and frequency of torture globally are not well described, which limits health care practitioners’ ability to adequately diagnose and treat the sequelae of torture.

**Objective:**

To rank the commonness of torture methods and identify the regions of the world with which they are associated.

**Data Sources:**

For this systematic review and meta-analysis, Ovid MEDLINE, Ovid Embase, Web of Science, and The Cochrane Library were searched from inception to July 2021.

**Study Selection:**

Included studies were peer-reviewed articles in English, contained an independent sample population of individuals who experienced torture, and outlined the type(s) of torture experienced. Excluded studies were not peer reviewed, lacked an independent sample population, or did not specify torture methods. Articles were chosen for inclusion by 2 independent and blinded reviewers, and a third, independent reviewer resolved discrepancies. Overall, 266 articles—15.3% of the 1739 studies initially identified for full review—met the inclusion criteria.

**Data Extraction and Synthesis:**

Data abstraction and quality assessment followed the Preferred Reporting Items for Systematic Reviews and Meta-Analyses guidelines. Data were extracted by 2 independent and blinded reviewers into predefined templates, and a third, independent reviewer resolved discrepancies. The risk of bias was evaluated using the Downs and Black Checklist.

**Main Outcomes and Measures:**

Torture methods were ranked by their average frequencies, numbers of reporting studies, and numbers of countries wherein the methods occurred.

**Results:**

A total of 9937 titles and abstracts were screened, and 266 studies encompassing 103 604 individuals (13 350 men, 5610 women, and 84 644 unspecified) were analyzed. Torture was reported for 105 countries; 21 methods accounted for 84% of all reported methods and 10 methods accounted for 78% of all physical tortures. The top 3 methods were beating or blunt-force trauma (reported in 208 studies and 59 countries; average frequency, 62.4%; 95% CI, 57.7%-67.1%), electrical torture (reported in 114 studies and 28 countries; average frequency, 17.2%; 95% CI, 15.0%-19.4%), and starvation or dehydration (reported in 65 studies in 26 countries; average frequency, 12.7%; 95% CI, 10.2%-15.2%). According to the Downs and Black appraisal tool, 50 studies were rated as good or excellent and 216 as fair or poor.

**Conclusions and Relevance:**

The findings of this study suggest that torture remains widespread. Although innumerable torture methods exist, a limited number account for the vast majority of reported tortures. So that targeted therapies may be developed, additional investigation is needed to better elucidate the sequelae associated with the most common torture methods, described here.

## Key Points

**Question:**

Which torture methods are most common, and where are they perpetrated?

**Findings:**

In this systematic review and meta-analysis of 266 studies with 103 604 individuals, torture was reported in 105 countries and a small number of methods (eg, beating or blunt-force trauma, electrical torture, starvation) accounted for the majority of the reported instances of torture.

**Meaning:**

In mapping the frequency and geographic distribution of torture methods, this study aims to help focus clinicians’ screening for torture sequelae in refugees.

## Teaser

This systematic review and meta-analysis ranks the commonness of torture methods and identifies the regions of the world with which they are associated.

## Introduction

The prohibition of torture is a fundamental principle of international law: 173 states have ratified the 1984 United Nations (UN) Convention against Torture.^1^ Nevertheless, Amnesty International has documented the practice of torture in at least 141 countries.^2^ Torture is defined by the World Medical Association (WMA) as “the deliberate, systematic or wanton infliction of physical or mental suffering by one or more persons acting alone or on the orders of any authority, to force another person to yield information, to make a confession, or for any other reason.”^3^

According to the UN Refugee Agency, 32% of the 63 190 submissions for resettlement in 2021 and 27% of the 116 481 submissions in 2022 were “survivors of violence and torture.”^4,5^ Because these figures include both violence and torture, it is difficult to estimate what proportion of the world’s nearly 100 million refugees have been tortured.^6^ Nevertheless, as war, climate change, and other global calamities continue to displace 44 000 individuals daily, the number of torture survivors seeking refuge in high-resource countries is rising.^6^ Health care practitioners globally are increasingly likely to encounter torture survivors in their clinical practices. These patients experience concurrent physical and psychological trauma that is often severe or debilitating.^7,8,9,10^ The methods, distribution, and frequency of torture globally are not well described. Understanding the most common methods of torture encountered by medical professionals—particularly in relation to country or region of origin—may assist in the diagnosis of physical and psychological trauma and can guide appropriate medical treatment of forcibly displaced people. To our knowledge, this systematic review and meta-analysis is the first to rank the commonness of specific torture methods utilized worldwide and identify the regions of the world with which they are associated.

## Methods

This systematic review and meta-analysis followed the Preferred Reporting Items for Systematic Reviews and Meta-Analyses (PRISMA) statement.^11^ The review was registered in PROSPERO, an international prospective register of systematic reviews, under registration identification number CRD42021270848 and published a priori.

### Data Sources

Searches in Ovid MEDLINE (All, 1946-2021), Ovid Embase (1974-2021), Web of Science, and The Cochrane Library (Wiley) were run from inception to July 27, 2021. In consultation with the other coauthors, the search strategy was designed by an experienced medical librarian at the Weill Cornell Medicine Samuel J. Wood library who holds a graduate degree in library sciences. The search strategy was additionally reviewed by a second medical librarian and included all appropriate controlled vocabulary and keywords for the concept of torture (eAppendix 1 in Supplement 1). The searches were not restricted by language, publication date, or article type.

### Study Selection

Retrieved studies were screened using Covidence (Veritas Health Innovation), an online software for systematic reviews. Titles and abstracts were reviewed against predefined inclusion and exclusion criteria by 2 independent reviewers (A.M., E.W., J.L., and A.L.), and a third, independent reviewer (A.M., E.W., and J.L.) resolved disagreements. The full texts for the included titles and abstracts were subsequently retrieved and screened for final inclusion by 2 independent reviewers (A.M., E.W., J.L., and A.L.), and disagreements were resolved by a third, independent reviewer (A.M., E.W., and J.L.). Articles considered for inclusion were peer reviewed (only article types requiring peer review were included), were full-text articles in English, contained an independent sample population of individuals who experienced torture as outlined by the WMA definition of torture, and reported the number of individuals who were subjected to at least 1 broad category (eg, physical, psychological, sexual, or sensory) or 1 specific method of torture. Among the internationally recognized definitions of torture, the WMA definition was chosen because it defines torture in the broadest terms and specifically encompasses torture perpetrated by gangs and militias.^12^ Excluded studies were not peer reviewed, lacked an independent sample population of individuals who experienced torture (eg, review papers, or the reported experiences did not satisfy the WMA definition for torture), could not be located (neither through any online source, nor through interlibrary loan), did not specify the method of torture, or contained a sample wherein individuals who experienced torture could not be clearly distinguished from those who did not. To ensure literature saturation, reference lists from the studies selected for inclusion were also searched and screened for eligibility.

### Data Extraction

A predefined, standardized template was created for data extraction (eTable 1 in Supplement 1). The first iteration of the investigated set of torture methods was taken from a previously reported list.^13^ Additional types of torture encountered during data collection were initially coded separately, and similar types or torture were subsequently consolidated into the final list of 45 individual torture methods (eTable 1 in Supplement 1). Two reviewers (A.M., E.W., J.L., and A.L.) independently extracted data from each article and a third, independent reviewer (A.M., E.W., and J.L.) resolved discrepancies. If an article provided data for multiple types of torture that fit the description for a single, predefined torture method, then the single largest number was retained for the torture method and, to avoid double counting, all of the other numbers were discarded. For example, an article may report that some number of individuals were punched, some were kicked, and some were beaten with the butt of a rifle. All 3 of these abuses are contained within the “beating of blunt-force trauma” torture method, so only the largest of the 3 numbers would be coded for the torture method.

### Statistical Analysis

The commonness of individual torture methods was estimated by 3 distinct measures: tallying the number of studies reporting the method, tallying the number of countries wherein the method was reported to occur (termed the minimal geographic extent), and averaging the reported frequencies of the method across all studies. The minimal geographic extent of a torture method was found by identifying all the countries wherein a method was clearly reported to have been perpetrated. If, for example, a study investigated individuals from multiple countries, but a torture method was applied to only part of the sample, then those countries could not be included in the method’s minimal geographic extent. The commonness of the torture methods was ranked by each measure and a final ranking was found through a consensus of the 3 distinct measures. Specifically, a torture method could be ranked among the top 10 most common methods only if the method was ranked in the top 10 by all 3 measures. As an additional check, the frequencies of the torture methods were also estimated by pooling individuals across all studies.

Conditional odds ratios calculated by Fisher exact test were used to evaluate the differences in the reported frequencies of torture methods between men and women who were subjected to torture. The conditional odds of men being subjected to a method was the number of clearly identified men exposed to the method divided by the number of men for whom the method was not reported, and the same for women. All hypothesis tests were 2-sided. Significance was set a priori at α < .05, and the Benjamini-Hochberg method was used to account for multiple hypothesis testing. Unless otherwise specified, means, average frequencies, and odds ratios are reported with 95% CIs; and median values are reported with IQRs. Associations between ratio variables were assessed by Pearson correlation coefficient.

The set of reported torture methods could vary considerably across articles. Examining only the torture methods does not, however, readily illuminate the degree of similarity between articles nor does it elucidate the source of the heterogeneity. For example, different sets of torture methods may be reported by distinct classes of articles. A pairwise similarity index (SI) was therefore devised to quantify the similarity between articles (mathematical definition in eAppendix 2 in Supplement 1). SI ranges from 0 to 1: 1 indicates perfect overlap in the reported methods and 0 indicates no overlap. The SI was then used to establish a hierarchy of similarity relationships between all pairs of articles; specifically, the studies were rearranged in an order that minimized their distances in the space of torture methods. Sorting the articles according to the hierarchy then permitted identification of clusters of similar studies. All analyses were performed in Matlab, version R2022b (MathWorks).

All included studies were subjected to critical appraisal using the Downs and Black Checklist. Each study’s reporting metrics, internal validity, external validity, and statistical power were appraised independently by 2 reviewers (E.W. and F.T.). Quality levels were assigned to each study (eTables 11 and 12 in Supplement 1) according to the categories proposed by Hooper et al^14^: excellent (Downs and Black score ≥26), good (Downs and Black score 20-26), fair (Downs and Black score 15-19), and poor (Downs and Black score ≤14).

## Results

A total of 9937 article titles and abstracts were screened, 1739 full-text articles were assessed, and 266 articles^7,12,15,16,17,18,19,20,21,22,23,24,25,26,27,28,29,30,31,32,33,34,35,36,37,38,39,40,41,42,43,44,45,46,47,48,49,50,51,52,53,54,55,56,57,58,59,60,61,62,63,64,65,66,67,68,69,70,71,72,73,74,75,76,77,78,79,80,81,82,83,84,85,86,87,88,89,90,91,92,93,94,95,96,97,98,99,100,101,102,103,104,105,106,107,108,109,110,111,112,113,114,115,116,117,118,119,120,121,122,123,124,125,126,127,128,129,130,131,132,133,134,135,136,137,138,139,140,141,142,143,144,145,146,147,148,149,150,151,152,153,154,155,156,157,158,159,160,161,162,163,164,165,166,167,168,169,170,171,172,173,174,175,176,177,178,179,180,181,182,183,184,185,186,187,188,189,190,191,192,193,194,195,196,197,198,199,200,201,202,203,204,205,206,207,208,209,210,211,212,213,214,215,216,217,218,219,220,221,222,223,224,225,226,227,228,229,230,231,232,233,234,235,236,237,238,239,240,241,242,243,244,245,246,247,248,249,250,251,252,253,254,255,256,257,258,259,260,261,262,263,264,265,266,267,268,269,270,271,272,273,274,275,276,277,278^ were included for analysis (eFigure 1 in Supplement 1). One article^274^ was published in 1947 and the rest in 1977 to 2021, with an average increase of 0.25 additional articles published per year from 1977 to 2021 (eFigure 2A in Supplement 1). A total of 103 604 individuals who experienced torture were identified (Table 1). The median (IQR) number of individuals who experienced torture per study was 25 (3-91), and the full range was 1 to 75 573. The median (IQR) number of reported torture methods was 6 (3-11), and the full range was 1 to 36 (Table 1; eFigure 2B in Supplement 1). Reports of torture could be identified for an average of 2.1 (95% CI, 1.6-2.6) countries per study, and the range was 0 to 39 countries. Only 1 country of torture could be identified for 143 studies (54%),^15,16,17,19,21,24,26,27,28,30,32,35,36,37,38,40,43,46,47,48,49,50,52,55,56,57,58,61,63,66,69,71,72,77,78,79,80,82,83,86,93,94,95,96,98,99,102,103,105,106,107,108,111,114,117,118,119,120,121,122,123,125,126,127,128,129,130,133,134,138,139,140,147,148,159,160,162,163,164,167,169,174,175,176,179,180,181,182,183,185,186,187,188,189,191,196,197,200,201,202,203,204,206,207,208,209,210,211,212,213,214,215,216,217,220,221,225,226,227,228,229,235,236,238,239,240,241,245,247,248,249,252,257,258,259,260,261,263,265,267,268,275,278^ 2 to 10 countries for 53 studies (20%),^41,42,54,65,67,74,75,89,92,104,109,112,113,115,124,137,141,142,143,144,145,146,149,153,154,155,156,158,166,168,171,173,192,194,198,199,205,222,223,230,232,233,234,237,243,246,251,262,269,270,272,274,277^ more than 10 countries for 11 studies (4%),^33,34,60,81,100,151,161,165,231,242,271^ and no single country of torture could be identified for 59 studies (22%).^7,12,18,20,22,23,25,29,31,39,44,45,51,53,59,62,64,68,70,73,76,84,85,87,88,90,91,97,101,110,116,131,132,135,136,150,152,157,170,172,177,178,184,190,193,195,218,219,224,244,250,253,254,255,256,264,266,273,276^ The location(s) of torture included regions encompassing multiple countries (ie, “Africa”) or parts of multiple countries (ie, “Kurdistan”) in 101 studies, and single countries could not be ascribed to those regions. Overall, 113 studies (43%)^7,16,17,22,23,27,30,31,33,34,40,41,43,45,46,49,51,55,57,58,59,60,61,63,64,65,67,70,71,72,76,77,79,82,84,86,89,90,91,94,96,98,100,104,106,108,109,110,112,113,114,116,118,123,128,132,134,137,140,142,146,148,149,150,156,157,159,161,166,169,170,172,173,177,178,181,182,185,186,189,192,194,197,198,199,201,202,205,206,209,210,211,216,221,223,226,228,229,231,232,233,234,235,242,244,251,253,256,259,261,269,272,277^ included men and women, 82 studies (31%)^18,20,25,26,28,29,35,37,38,39,44,47,48,52,54,56,62,66,68,69,83,87,93,97,99,102,103,105,107,111,117,119,120,127,130,133,139,151,152,154,162,163,164,167,171,174,175,179,183,188,190,191,193,195,200,203,204,207,212,213,217,218,219,220,222,224,227,238,240,245,248,250,252,254,255,258,264,267,268,274,275,276^ included only men, 27 studies (10%)^19,21,32,36,42,50,53,80,81,85,92,95,101,125,135,136,141,143,145,147,155,165,180,196,247,266,271^ included only women, and the gender of the sample could not be determined for 44 studies (17%)^12,15,24,73,74,75,78,88,115,121,122,124,126,129,131,138,144,153,158,160,168,176,184,187,208,214,215,225,230,236,237,239,241,243,246,249,257,260,262,263,265,270,273,278^ (Table 1). Genders could clearly be determined for 13 350 men and for 5610 women. Among the 113 studies that included men and women, there were more men than women in 88 studies (78%),^16,22,27,30,31,33,34,40,41,43,46,49,51,55,57,58,59,60,61,63,64,65,67,70,71,72,76,77,79,90,94,96,98,100,104,106,108,109,112,116,118,123,128,132,134,137,146,150,156,157,159,161,166,169,170,172,173,177,182,185,186,192,194,197,198,199,201,202,206,209,210,211,221,223,226,228,229,231,232,234,242,244,253,259,261,269,272,277^ equal numbers of men and women in 9 studies (8%),^23,84,86,91,110,178,205,216,235^ and more women than men in 16 studies (14%).^7,17,45,82,89,113,114,140,142,148,149,181,189,233,251,256^ Moreover, these 113 studies included an average of 2.6 (95% CI, 2.1-3.2) times as many men as women on average. eTable 2 in Supplement 1 lists individual characteristics of the included articles.

**Table 1.** Study Demographics

Characteristic	Studies, No. (%) (N = 266)
No. of tortured individuals per study	
1-9	94 (35.3)
10-99	112 (42.1)
100-999	55 (20.7)
1000-9999	4 (1.5)
≥10 000	1 (0.4)
No. of reported torture methods per study	
1-10	198 (74.4)
11-20	58 (21.8)
21-30	7 (2.6)
≥31	3 (1.1)
No. of countries where torture was reported per study	
1	143 (53.8)
2-10	53 (19.9)
>10	11 (4.1)
Country of torture could not be determined^a^	59 (22.2)
Gender(s) specified	
Men and women	113 (42.5)
Only men	82 (30.8)
Only women	27 (10.2)
Gender of could not be determined^b^	44 (16.5)
Gender of individuals across all articles, No./total No. (%)	
Men	13 350/103 604 (12.9)
Women	5610/103 604 (5.4)
Unspecified	84 644/103 604 (81.7)

^a^ No country of torture was specified or only a multicountry region was specified.

^b^ The gender of the participants was not specified for the study’s entire sample or for the subsample that could be included for analysis.

Of the 45 individual torture methods assessed, 38 methods (84%) were experienced by at least 1% of individuals on average, 25 (56%) by 5% or more, 11 (24%) by 10% or more, and 2 (4%) by 20% or more. Summing the average frequencies for all 45 torture methods produced an estimate for the average number of torture methods experienced by each individual (eAppendix 3 in Supplement 1). This calculation yields an average of 3.6 (95% CI, 2.6-4.6) torture methods per individual. A total of 37 torture methods (82%) were reported by 10 or more studies, 32 (71%) by 20 or more, 18 (40%) by 50 or more, and 3 (7%) by 100 or more (eFigure 3 in Supplement 1).

Using the average frequencies of the torture methods, number of studies reporting the methods, and number of countries wherein the methods were perpetrated, the torture methods were ranked, and a set of the most common torture methods was identified (eTable 3 and eFigure 3 in Supplement 1). The list contains methods from each broad category of torture: physical, psychological, sexual, and sensory. Physical torture was the most commonly reported category and accounted for approximately half of all the tortures reported. Individuals were subjected to an average of 2.0 (95% CI, 1.5-2.5) types of physical torture, 1.2 (95% CI, 1.0-1.4) types of psychological torture, 0.3 (95% CI, 0.2-0.4) types of sexual torture, and 0.1 (95% CI, 0.0-0.2) types of sensory torture. The top 3 methods were beating or blunt-force trauma (reported in 208 studies and 59 countries; average frequency, 62.4%; 95% CI, 57.7%-67.1%), electrical torture (reported in 114 studies and 28 countries; average frequency, 17.2%; 95% CI, 15.0%-19.4%), and starvation or dehydration (reported in 65 studies in 26 countries; average frequency, 12.7%; 95% CI, 10.2%-15.2%). Ranking the physical torture methods according to the same 3 strategies generated a list of the top 10 physical torture methods, which account for 78% of the reported physical tortures (Table 2). Ranking the physical torture methods according to their pooled frequencies generated the same top 10 list, although in a slightly different order (eTable 4 in Supplement 1).

**Table 2.** Top 10 Physical Torture Methods as Ranked by a Consensus of 3 Strategies

Physical torture methods^a^	Average frequency, % (95% CI)	No. of studies	No. of countries
Beating or blunt-force trauma	62.4 (57.7-67.1)	208	59
Electrical torture	17.2 (15.0-19.4)	114	28
Starvation or dehydration	12.7 (10.2-15.2)	65	26
Foot whipping	12.6 (11.6-13.6)	65	23
Suspension	11.0 (8.3-13.7)	75	21
Asphyxiation or suffocation	8.9 (5.6-12.2)	68	21
Binding or restricted movement	8.3 (5.9-10.7)	51	21
Other forced positions	8.0 (7.0-9.0)	57	18
Sharp objects or penetrating trauma	7.8 (4.1-11.5)	65	24
Burning	6.9 (6.3-7.5)	84	25

^a^ The listed physical torture methods all ranked among the top 10 physical methods by all 3 ranking strategies.

**Figure 1.**
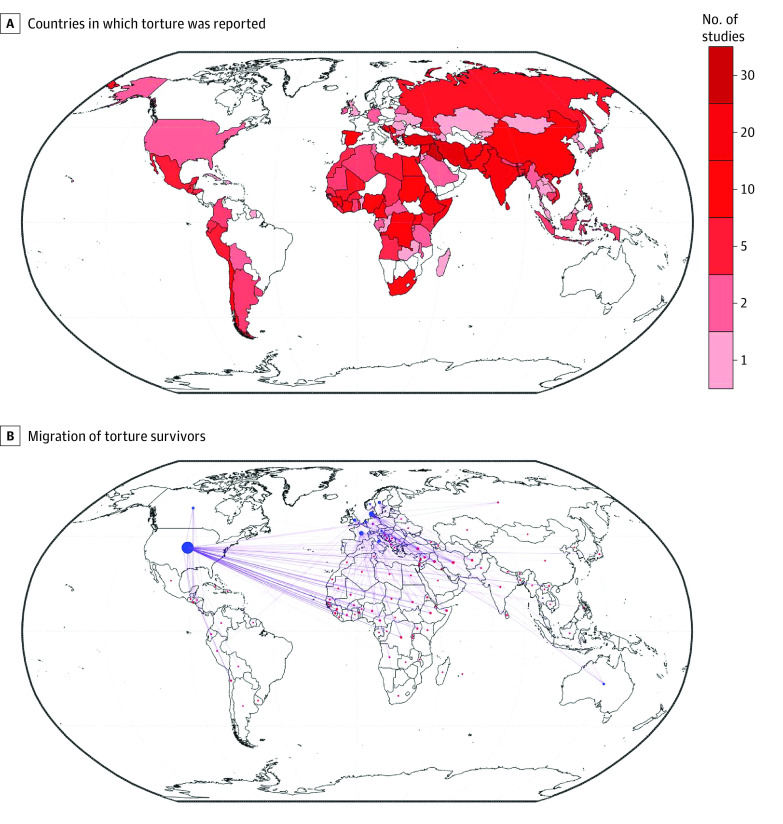
Minimal Geographic Extent of Torture and the Migration of Torture Survivors B, Red and blue dots indicate where individuals were tortured and encountered for study, respectively. The size of the dots indicates the number of studies for which individuals reported torture or were encountered for study. Each purple line designates a single migratory pathway. Multiple pathways may arise from a single study. Only those studies that listed a single encounter location and listed locations of torture were used to generate the map, and locations of encounters that coincided with locations of torture are not shown. Consequently, data from 180 studies were included in the map. eTable 8 in Supplement 1 lists the countries wherein individuals were encountered for study.

Torture was geographically widespread. Individuals reported that they were subjected to torture in 105 countries (Figure 1A; eTable 5 in Supplement 1) and in 18 of 22 UN subregions (eFigure 4A and eTables 6-8 in Supplement 1). Each category of torture was also widely distributed. Physical torture was clearly reported for 67 countries and by 253 studies,^7,12,15,16,17,18,20,21,22,23,24,25,26,27,28,29,30,31,33,34,35,36,37,38,39,40,41,42,43,44,45,46,47,48,49,50,51,52,53,54,55,56,57,58,59,60,61,62,63,64,65,66,67,68,69,70,71,72,73,74,75,76,77,78,79,80,81,82,83,84,85,86,87,88,90,91,92,93,94,95,96,97,98,99,100,101,102,103,104,105,106,107,108,109,110,111,112,113,114,115,116,117,118,119,120,121,122,123,124,125,126,127,128,129,130,131,132,133,134,135,136,137,138,139,141,142,143,145,146,147,148,149,150,151,152,153,154,155,156,157,159,160,161,162,163,166,167,168,169,170,171,172,173,174,175,176,177,178,179,180,181,182,183,184,185,186,187,188,189,190,191,192,193,194,195,196,197,198,199,200,201,202,203,204,205,206,207,208,209,210,211,212,213,214,215,216,218,219,220,221,222,223,224,225,226,227,228,229,230,231,232,233,234,235,236,237,238,239,240,241,242,243,244,247,248,249,250,251,252,253,254,255,256,257,258,259,260,261,262,263,265,266,267,268,269,270,272,273,274,275,276,277,278^ psychological torture for 62 countries and by 188 studies,^12,16,17,18,19,20,22,23,24,28,30,32,33,34,36,38,40,41,42,43,45,46,48,53,54,55,56,57,58,59,61,62,63,65,66,67,72,73,76,78,79,80,81,82,83,84,86,87,88,90,91,92,94,95,98,99,100,101,103,104,106,109,110,111,112,113,114,115,116,117,118,119,120,121,122,123,124,125,126,127,128,130,131,133,134,135,136,137,138,139,141,143,144,145,146,147,148,149,150,151,153,154,155,157,159,160,166,167,168,169,170,171,173,175,176,177,178,179,181,182,183,184,185,186,187,188,191,192,193,194,196,197,198,199,202,203,205,206,208,209,210,211,214,215,216,217,219,221,225,226,228,230,232,233,234,235,236,237,239,241,242,243,245,246,249,250,251,252,253,256,257,258,259,260,261,262,263,264,265,266,267,268,269,270,271,273,277,278^ sexual torture for 46 countries and by 147 studies,^7,12,16,19,22,23,27,28,30,33,34,38,39,40,41,45,49,51,55,58,59,61,63,64,65,68,69,70,71,72,73,74,75,78,79,81,82,86,89,90,91,92,94,96,99,100,101,106,108,109,110,112,113,114,115,116,117,121,122,124,126,128,130,131,133,134,136,137,140,141,142,143,145,147,148,149,150,151,154,155,158,161,164,165,166,168,169,170,173,175,180,181,182,183,184,185,189,191,192,194,195,196,197,198,199,202,203,205,206,208,209,210,211,215,216,221,225,226,228,230,231,232,233,235,237,239,241,242,243,248,250,251,253,254,256,257,258,261,262,265,266,268,270,271,272,275,277^ and sensory torture for 25 countries and by 61 studies^17,22,38,40,41,43,48,51,58,63,65,72,79,81,83,89,90,99,106,109,110,114,115,122,123,124,131,133,134,137,147,149,150,161,170,175,176,177,181,183,184,188,193,197,198,205,212,217,221,225,230,231,232,241,251,257,258,260,262,270,277^ (eFigures 4B-4E in Supplement 1).

Migration trends were identified by comparing the countries wherein individuals were subjected to torture with the countries wherein the same individuals were encountered for study (Figure 1B; eTables 5 and 9 in Supplement 1). Migration of at least part of the study sample was found for 189 articles (71%),^12,18,20,21,22,23,24,25,28,31,32,33,34,36,37,38,39,41,42,45,47,49,50,51,53,54,55,56,59,60,61,62,63,64,65,67,68,69,70,71,73,74,75,76,78,79,80,81,83,84,85,86,88,89,91,92,93,94,98,99,100,101,102,104,105,106,107,108,109,110,111,112,113,114,115,116,117,118,120,121,122,124,127,130,132,133,134,136,137,138,141,142,143,144,145,146,148,149,150,151,152,153,154,155,156,157,158,160,161,165,166,167,168,169,170,171,173,174,175,176,177,178,180,181,182,183,184,191,192,193,194,195,197,198,199,202,203,204,205,212,213,214,215,216,217,221,222,223,224,226,229,230,231,232,233,234,235,237,238,240,241,242,243,244,245,246,249,250,251,252,253,254,255,256,257,259,260,261,262,264,267,269,270,271,272,274,275,276,277^ and most of these articles accessed individuals who experienced torture who migrated to the United States, Canada, Australia, and parts of Europe. Individuals were encountered for study in the same country that they were subjected to torture for 73 articles (27%).^15,16,17,19,26,27,30,35,40,43,46,48,52,57,58,66,72,77,82,95,96,100,103,112,119,123,125,126,128,129,139,140,145,146,147,154,155,159,162,163,164,168,179,185,186,187,188,189,196,200,201,206,207,208,209,210,211,220,225,227,228,231,232,236,239,247,248,258,262,263,265,268,278^ The researchers who conducted the studies were housed by 44 countries (eFigure 5 and eTable 10 in Supplement 1). The greatest number of studies came from the United States, followed by Denmark, the United Kingdom, Canada, and Turkey.

Except for stoning and sexual enslavement (reported for 0 and 1 country, respectively), every torture method was reported to have occurred in 3 or more countries: 38 torture methods (84%) were perpetrated in 5 or more countries, 32 (71%) in 10 or more countries, 19 (42%) in 20 or more countries, and 4 (9%) in 40 or more countries. It was uncommon for individual torture methods to be regionally localized. Excluding stoning, which could not be ascribed to any UN subregion, sexual enslavement was reported for just 1 UN subregion and muscle crushing with roller (*ghotna*) was reported for just 2 neighboring UN subregions located within the same continent. The remaining 42 methods (93%) were reported for 3 or more subregions that spanned 2 or more continents. Specifically, 35 methods (78%) were reported for 5 or more subregions, and 19 methods (42%) were reported for 10 or more subregions. Some regional differences were found for individual torture methods (eFigure 6 in Supplement 1).

**Figure 2.**
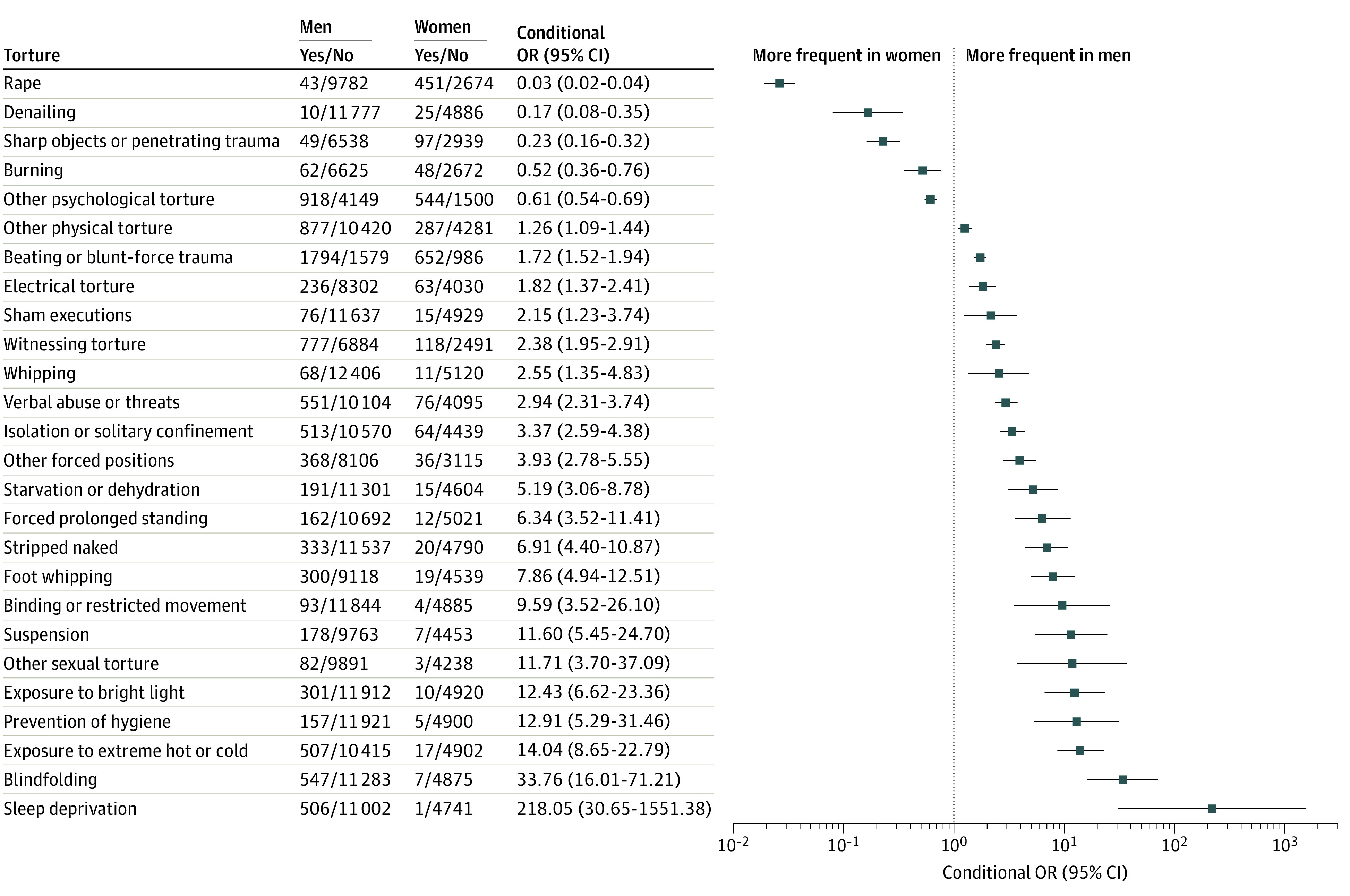
Differences in Torture-Method Frequencies Between Men and Women A forest plot depicts the conditional odds ratios (ORs) and 95% CIs for the torture methods that were reported more frequently for 1 gender. For every listed torture method, *P* < .006. Torture methods that were applied to only 1 gender had infinite odds ratios and are not included, ie, female genital mutilation or cutting was the only torture method that was reported only for women; gunshot, muscle crushing with roller (*ghotna*), deprivation of medical care, pharmacological torture, loud noises, and genital trauma were clearly reported only for men.

**Figure 3.**
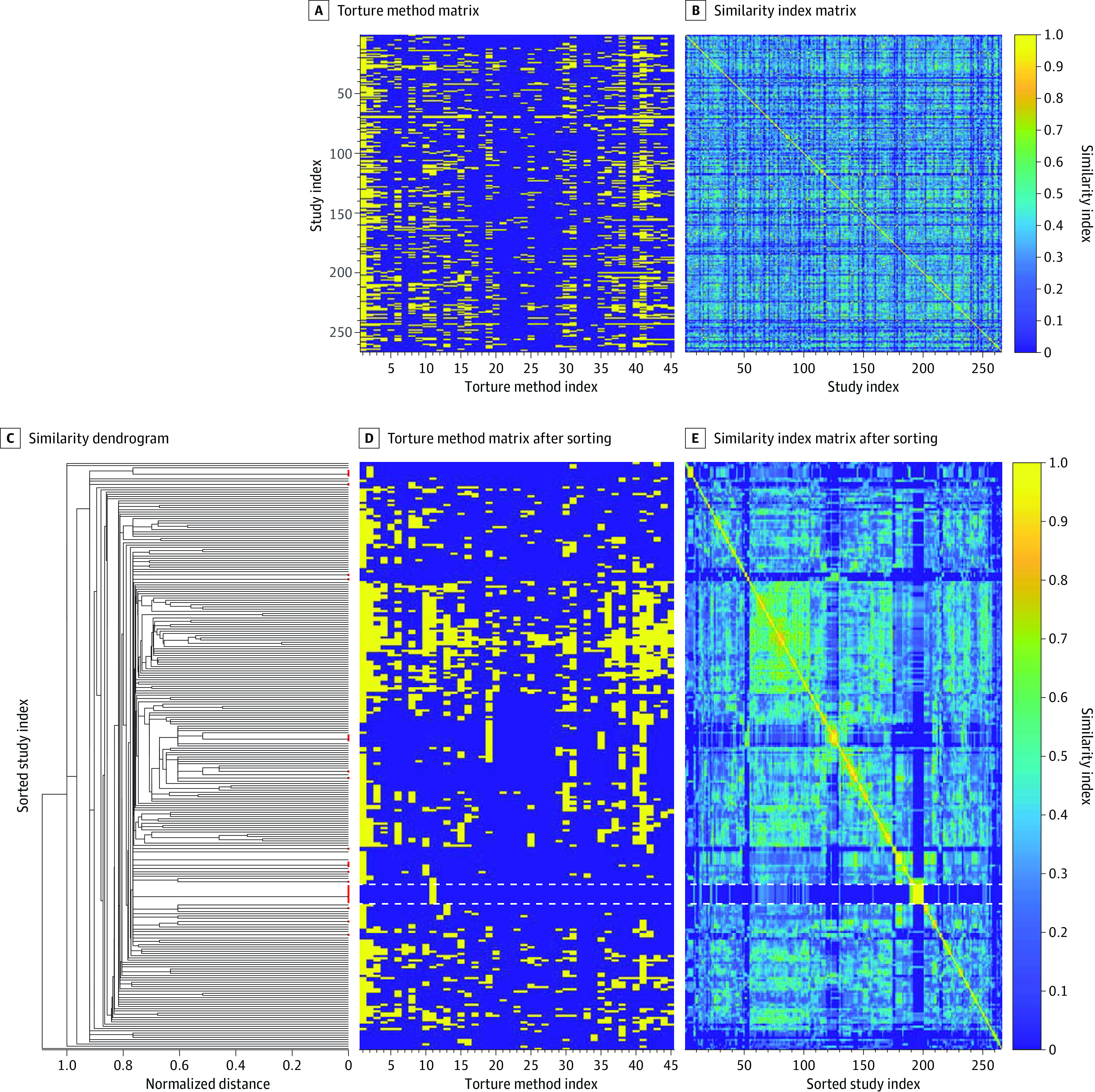
Similarity Between Studies A, A matrix displays the torture methods that were reported by each study: a yellow stripe indicates that the torture method was reported by the individual study. eTable 2 in Supplement 1 lists the torture method that corresponds with each method’s index. B, A heat map portrays the degree of similarity between each pair of studies. A higher similarity index indicates greater overlap in the number of torture methods reported by both studies: a similarity index of 1.0 (yellow) indicates perfect overlap in the torture methods reported by both studies and a similarity index of 0.0 (dark blue) indicates no overlap. As shown, the data do not permit identification of clusters of similar studies. C, A dendrogram shows the normalized distance—in the space of torture methods—between studies, indicated by the location of the vertical line that joins 2 or more studies. The branches of the dendrogram are arranged in an order that minimizes the distance between clusters. Red lines identify clusters wherein the reported torture methods overlap perfectly among the studies contained within the cluster. D and E, As for panels A and B, but after reordering the studies according to the dendrogram. After reordering the studies, several small clusters emerge. For example, the dashed, white lines identify a cluster of 9 studies that reported only foot whipping. Considerable heterogeneity exists, however, across the entire sample of studies. To allow for better visualization of the dendrogram, panels D and E are stretched vertically relative to panels A and B.

Although no difference was found for the average number of torture methods reported by men and women (4.0 [95% CI, 2.0-6.1] and 3.5 [95% CI, 0.5-6.5] methods, respectively), 6 methods were reported more often by women and 27 methods were reported more often by men (Figure 2). With regard to the reported torture methods, the 266 included articles were highly heterogeneous (Figure 3). There were 237 unique collections of torture methods reported. The similarity index (SI) between 2 articles provides a measure of the proportion of torture methods that are common to both articles and that is scaled to the number of methods reported in both articles. The median (IQR) SI for all pairs of articles was 0.29 (0.16-0.42). The SI was 0.50 or greater for 4724 pairs of articles (13.4%) and 0.80 or greater for 130 pairs (0.4%) (eFigure 7A in Supplement 1). The median (IQR) number of overlapping torture methods was 2 (1-3), and the range was 0 to 34 (eFigure 7B in Supplement 1). Of the 35 245 pairs of articles, there was no overlap in the reported torture methods for 7281 pairs of articles (20.7%). Just 69 pairs (0.2%) reported identical collections of torture methods, which encompassed 44 articles^21,24,25,26,29,37,45,47,52,77,97,100,102,105,107,112,113,128,132,152,156,157,158,164,168,178,192,201,218,222,223,224,227,237,238,240,244,246,247,255,264,267,274,276^ in 15 clusters: 1 cluster of 9 articles (that reported only foot whipping),^26,29,107,156,222,223,224,244,255^ 1 of 5 articles (that reported only beating or blunt-force trauma),^152,201,240,247,276^ 2 clusters of 4 articles,^25,37,45,47,100,112,128,238^ and 11 clusters of 2 articles each.^24,52,77,97,102,105,113,132,157,158,164,168,178,192,218,227,237,246,264,267,274^ Less well-defined clusters were also present, including a cluster of 50 articles whose similarity arose from reporting a large number of torture methods.

According to the Downs and Black tool, the quality of 2 studies (0.8%)^132,260^ was rated as excellent, 48 (18.1%)^7,12,27,30,40,43,54,58,59,61,63,69,71,73,83,95,96,99,101,105,106,126,128,133,144,150,155,157,158,160,174,175,176,189,192,198,199,210,223,224,230,232,241,251,261,265,270,271^ as good, 76 (28.6%)^24,29,33,34,48,49,51,60,65,70,74,75,76,81,88,90,92,98,108,109,111,112,113,114,115,116,117,118,120,121,123,124,130,142,146,149,152,166,168,171,172,173,178,179,184,185,186,190,194,197,201,209,211,214,215,221,222,226,229,233,238,243,244,253,255,256,257,259,262,264,267,272,273,277^ as fair, and 140 (52.6%)^15,17,18,19,20,21,22,25,26,28,31,32,35,36,37,38,39,41,42,44,45,46,47,50,52,53,55,56,57,62,64,66,67,68,72,77,78,79,80,82,84,85,86,87,89,91,93,94,97,100,102,103,104,107,110,119,122,125,127,129,131,134,135,136,137,138,139,140,141,143,145,147,148,151,153,154,156,159,161,162,163,164,165,167,169,170,177,180,181,182,183,187,188,191,193,195,196,200,202,203,204,205,206,207,208,212,213,216,217,218,219,220^ as poor (eTable 11 in Supplement 1). Whereas clear study objectives, clear descriptions of the participants included in the study, clear discussion of findings and outcomes, and descriptions of adverse events consequential to the study intervention were frequently present in the articles, the following Downs and Black Checklist items were represented less frequently: probability values, appropriate statistical testing, sufficient power to detect important effects, internal and external validity, representative samples, a description of confounding variables, and characteristics of individuals lost to follow-up (eTable 12 in Supplement 1).

## Discussion

To our knowledge, this is the first, large-scale systematic review that ranks the commonness of torture methods worldwide. Notwithstanding the existence of innumerable torture methods, 21 methods accounted for 84% of the reported instances of torture and 10 methods accounted for 78% of the reported instances of physical torture. This study confirms that torture is pervasive and practiced in nearly every region of the world: the 266 included articles—published from 1947 onward—identified instances of torture in 105 countries (within 18 of 22 UN subregions). The articles identified 103 604 individuals who were subjected to torture and demonstrated that perpetrators usually use multiple methods in torturing an individual (conservative estimate of an average of 3.6 [95% CI, 2.6-4.6] methods per person), most often across multiple categories: the individuals in this study were subjected to an average 2 types of physical torture and 1 type of psychological torture; around 1 in 4 individuals were subjected to sexual torture and roughly 1 in 9 to sensory torture.

By mapping the geographic distributions for torture methods, we aim to aid clinicians’ approach to caring for asylum seekers and refugees. Knowing, for example, that a torture survivor emigrated from India should prompt clinicians to ask about exposure to muscle crushing with roller (*ghotna*) in addition to screening for more ubiquitous torture methods. Despite small regional differences, substantial commonalities are suggested by our estimates for the frequency with which individuals were subjected to each torture method: That just several methods account for the vast majority of the reported instances of torture implies that most individuals are subjected to some combination of the common methods. Although the experience of each torture survivor is unique, common threads exist among the kinds of torture that are perpetrated, which permits meaningful research in what would otherwise be considered a highly heterogeneous population.

The identified migration trends indicate that the published medical literature captures primarily refugees and asylum seekers. Data from the UN High Commissioner for Refugees and the Armed Conflict Location and Event Data Project, which document sources of forcible displacement, suggest that individuals are likely being tortured in more regions than represented in the published literature.^6,279^ Internally displaced persons and stateless individuals—populations that have largely been inaccessible to researchers—contribute considerably to this gap, signaling that additional research is needed to reach these vulnerable groups.

Whereas accurate figures for the numbers of men and women who were subjected to torture worldwide are lacking, global reports estimate that men and women are forcibly displaced in roughly equal numbers.^6^ We do not expect that men are tortured at disproportionately greater rates than women. The gender imbalance in our sample likely underscores a deficiency in researchers’ ability to access female torture survivors and an assumption that torture is more commonly perpetrated against men. Research focusing on women who were subjected to torture is needed. Similar numbers of torture methods were applied to men and women on average. Although many methods were reported more frequently for 1 gender, both men and women were subjected to each of the top torture methods. Rape, for example, was reported far more frequently for women, but some men were also raped. Because the sequelae of torture are frequently missed,^7^ clinicians must take special care to avoid gender biases in evaluating torture survivors. The standard of care for refugees and forcibly displaced individuals must include questions about exposure to torture, and both men and women should be screened for symptoms arising from physical, psychological, sexual, and sensory torture.

Analyzing the SI revealed considerable heterogeneity in the articles’ reporting of torture methods and also identified a few clusters of studies devoted to particular torture methods, such as foot whipping. Dedicating several studies to a specific torture method engenders a depth of evidence that affords better understanding of the sequelae arising from that method and enables the development of targeted therapies. Researchers have, for example, identified chronic neuropathic pain as a sequela of foot whipping, permitting pharmacological management of symptoms.^280^ Evidence-based understanding of the sequelae arising from most torture methods is lacking, and deeper investigations into the common methods are needed to further the standard of care in refugee health.

### Limitations

Several limitations warrant discussion. Owing to multiple sources of underreporting, we likely underestimate the true frequencies and geographic extents of the evaluated torture methods: few studies endeavored to catalog torture methods in detail; abuses that constitute torture—including widespread practices like sexual enslavement, forced marriage, forced pregnancy, and police violence—are not universally recognized and reported as such; researchers are unable to access certain tortured populations; and individuals who experienced torture—for a variety of reasons, including fears arising from medical professionals’ complicity in torture—may not disclose all of their experiences to researchers. Moreover, specific torture methods could not be ascribed to every country for which torture was reported. Researchers may also believe that torture occurs almost exclusively in regions with few human rights protections, which could bias investigative efforts toward specific nations. The exclusion of non-English articles (7% of the articles reviewed at the full-text stage) may engender further underestimation of the true burden of torture. The search also excluded books and other materials that were not peer reviewed, sources that might contain important data sets. Our findings should be considered together with other international surveys to fully appreciate the scope of torture around the world.

The broad range of sample sizes and the varied objectives—such as focusing on a single torture method and ignoring all others—among the included articles present additional limitations. Averaging frequencies across studies and using alternative ranking measures (specifically, the numbers of studies and countries reporting a torture method) limits the degree by which a few large studies could skew the results but strengthens the relative contribution from small studies. Calculating frequencies by pooling data—a strategy whose bias is inverse that of averaging—generated exactly the same lists of the top ten physical tortures, albeit in a different order.

Because the time at which torture occurred was unclear for many articles, we cannot comment on how the frequencies of torture methods may have changed over time. The search was also not designed to identify and rank the entities that perpetrate torture. Both topics warrant further investigation; answering these questions may inform international policies aiming to diminish the practice of torture around the world.

## Conclusions

By delineating the most common torture methods and mapping regions within which torture methods are practiced, we can begin to better understand the experiences of refugees who have been tortured. This work is, however, incomplete: a system that tracks the global occurrence of torture, comprehensively delineates the methods used, and identifies the responsible perpetrators is urgently needed. It will also be important to establish causes of perpetrator impunity, particularly in the case of state actors, and investigate opportunities for prevention. To ensure that adequate care is accessible to this vulnerable population, future research should additionally aim to better correlate torture methods with their physical and psychological sequelae, to develop diagnostic tools, and to design effective treatment pathways.

## Notes

Supplement 1. **eFigure 1.** PRISMA Flow Diagram and Study Selection **eAppendix 1.** Search Strategies for Databases **eAppendix 2.** Mathematical Definition and Explanation of the Similarity Index (SI) **eAppendix 3.** The Calculation for the Average Total Number of Torture Methods per Individual **eTable 1.** Categories for the Data Extraction Form **eFigure 2.** Publication Years and Distribution of Reported Torture Methods for the Included Studies **eTable 2.** Characteristics of Included Studies **eTable 3.** Top Torture Methods as Ranked by a Consensus of 3 Strategies **eFigure 3.** Ranking of All Torture Methods **eTable 4.** Top 10 Torture Methods Estimated by Pooled Frequencies **eTable 5.** Countries in Which Individuals Reported That Torture Occurred **eFigure 4.** UN Subregions in Which Torture Occurred and Minimal Geographic Extent of Torture Categories **eTable 6.** UN Subregions in Which Individuals Reported That Torture Occurred **eTable 7.** Countries Where Torture Mechanisms Were Reported to Occur, Organized by Country **eTable 8.** Countries Wherein Each Torture Method Was Reported to Occur, Organized by Torture Method **eTable 9.** Countries in Which Individuals Who Experienced Torture Were Encountered for Study **eFigure 5.** Countries Hosting Researchers **eTable 10.** Countries Hosting Researchers **eFigure 6.** Regional Variation for the Top 3 Torture Methods **eFigure 7.** Distributions for the Similarity Index and the Number of Overlapping Torture Methods **eTable 11.** Assessment of Risk of Bias Using the Downs and Black Checklist **eTable 12.** Number of Articles Satisfying Each Downs and Black Checklist Item

Supplement 2.
Data Sharing Statement
