# Errors in Author Affiliations

**DOI:** 10.1001/jamanetworkopen.2023.44501

**Published:** 2023-11-06

In the Original Investigation titled “Reported Methods, Distributions, and Frequencies of Torture Globally: A Systematic Review and Meta-Analysis,”^1^ published on October 3, 2023, there were errors in the author affiliations. Dr Pilato’s affiliation should have been Temple University Hospital, Philadelphia, Pennsylvania. Ms Jedlicka’s affiliation should have been Samuel J. Wood Library, Weill Cornell Medicine, New York, New York. This article has been corrected.^1^
