# Supplementary material for: Reported Methods, Distributions, and Frequencies of Torture Globally: A Systematic Review and Meta-Analysis
Source: JAMA Netw Open. 2023 Oct 3;6(10):e2336629. doi: 10.1001/jamanetworkopen.2023.36629 (PMC10548313; doi:10.1001/jamanetworkopen.2023.36629)
Supplement: Supplement 1. — eFigure 1. PRISMA Flow Diagram and Study Selection eAppendix 1. Search Strategies for Databases eAppendix 2. Mathematical Definition and Explanation of the Similarity Index (SI) eAppendix 3. The Calculation for the Average Total Number of Torture Methods per Individual eTable 1. Categories for the Data Extraction Form eFigure 2. Publication Years and Distribution of Reported Torture Methods for the Included Studies eTable 2. Characteristics of Included Studies eTable 3. Top Torture Methods as Ranked by a Consensus of 3 Strategies eFigure 3. Ranking of All Torture Methods eTable 4. Top 10 Torture Methods Estimated by Pooled Frequencies eTable 5. Countries in Which Individuals Reported That Torture Occurred eFigure 4. UN Subregions in Which Torture Occurred and Minimal Geographic Extent of Torture Categories eTable 6. UN Subregions in Which Individuals Reported That Torture Occurred eTable 7. Countries Where Torture Mechanisms Were Reported to Occur, Organized by Country eTable 8. Countries Wherein Each Torture Method Was Reported to Occur, Organized by Torture Method eTable 9. Countries in Which Individuals Who Experienced Torture Were Encountered for Study eFigure 5. Countries Hosting Researchers eTable 10. Countries Hosting Researchers eFigure 6. Regional Variation for the Top 3 Torture Methods eFigure 7. Distributions for the Similarity Index and the Number of Overlapping Torture Methods eTable 11. Assessment of Risk of Bias Using the Downs and Black Checklist eTable 12. Number of Articles Satisfying Each Downs and Black Checklist Item [file jamanetwopen-e2336629-s001.pdf]

Supplemental Online Content

Milewski A, Weinstein E, Lurie J, et al. Reported methods, distributions, and frequencies of torture globally. *JAMA Netw Open.* 2023;6(10):e2336629. doi:10.1001/jamanetworkopen.2023.36629

eFigure 1. PRISMA Flow Diagram and Study Selection

eAppendix 1. Search Strategies for Databases

eAppendix 2. Mathematical Definition and Explanation of the Similarity Index (SI)

eAppendix 3. The Calculation for the Average Total Number of Torture Methods per Individual

eTable 1. Categories for the Data Extraction Form

eFigure 2. Publication Years and Distribution of Reported Torture Methods for the Included Studies

eTable 2. Characteristics of Included Studies

eTable 3. Top Torture Methods as Ranked by a Consensus of 3 Strategies

eFigure 3. Ranking of All Torture Methods

eTable 4. Top 10 Torture Methods Estimated by Pooled Frequencies

eTable 5. Countries in Which Individuals Reported That Torture Occurred

eFigure 4. UN Subregions in Which Torture Occurred and Minimal Geographic Extent of Torture Categories

eTable 6. UN Subregions in Which Individuals Reported That Torture Occurred

eTable 7. Countries Where Torture Mechanisms Were Reported to Occur, Organized by Country

eTable 8. Countries Wherein Each Torture Method Was Reported to Occur, Organized by Torture Method

eTable 9. Countries in Which Individuals Who Experienced Torture Were Encountered for Study

eFigure 5. Countries Hosting Researchers

eTable 10. Countries Hosting Researchers

eFigure 6. Regional Variation for the Top 3 Torture Methods

eFigure 7. Distributions for the Similarity Index and the Number of Overlapping Torture Methods

eTable 11. Assessment of Risk of Bias Using the Downs and Black Checklist

eTable 12. Number of Articles Satisfying Each Downs and Black Checklist Item

This supplemental material has been provided by the authors to give readers additional information about their work.

© 2023 Milewski A et al. *JAMA Network Open.*

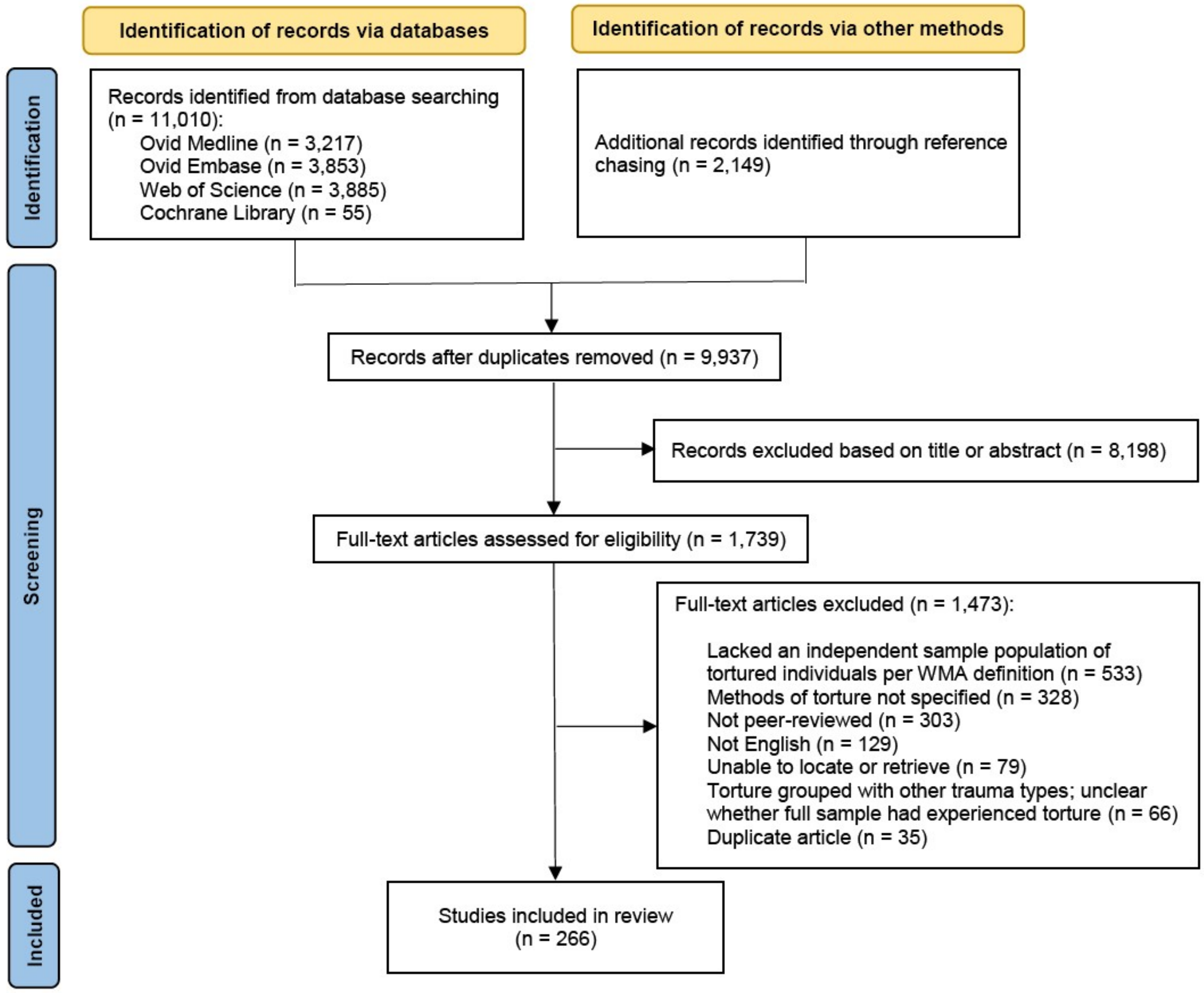

eFigure 1. PRISMA Flow Diagram and Study Selection

© 2023 Milewski A et al. *JAMA Network Open.*

eAppendix 1. Search Strategies for Databases

Ovid MEDLINE (R) ALL: 1946 to July 27, 2021
1. torture/
2. tortur*.tw.
3. 1 or 2

Ovid Embase: 1974 to 2021 July 27
1. torture/ or torture survivor/
2. tortur*.tw.
3. 1 or 2

Web of Science:
1. tortur*[ti]

Wiley Cochrane Library:
1. (tortur*):ti,ab
2. MeSH descriptor: [Torture] this term only
3. #1 or #2

© 2023 Milewski A et al. *JAMA Network Open.*

eAppendix 2. Mathematical Definition and Explanation of the Similarity Index (SI)

A pairwise "similarity index" (SI) was devised to evaluate the heterogeneity among the articles. For each study, a 45-dimensional, binary torture vector was constructed wherein at each position a "1" indicated that the corresponding method was reported by the study and a "0" indicated that the method was not reported. The torture vectors were normalized to unit length. The dot product between two torture vectors produced the SI between the two corresponding studies. The SI is equal to the geometric mean of the proportions of overlapping methods for two studies:

$$SI_{ij} = \sqrt{\left(\frac{k_{ij}}{n_i}\right)\left(\frac{k_{ij}}{n_j}\right)} = \frac{k_{ij}}{\sqrt{n_i}\sqrt{n_j}}$$

k_{ij} represents the number of torture methods that studies i and j have in common, and n_i and n_j are the total number of methods reported by studies i and j, respectively. SI ranges from zero to one: one indicates perfect overlap in the reported methods and zero indicates no overlap. To identify clusters of similar studies, a hierarchical tree was constructed in an order that minimized the distances between the studies' normalized torture vectors.

eAppendix 3. The Calculation for the Average Total Number of Torture Methods per Individual

Let x_{ij} give the number of individuals in study *i* subjected to method *j*, let n_i give the total number of individuals in study *i*, and let *N* give the total number of studies. For study *i*, the average number of tortures reported per individual is found by adding up all the tortures and dividing by the number of individuals in the study. Let T_i give the average number of tortures reported per individual in study *i*:

$$T_i = \frac{1}{n_i}\sum_j x_{ij}.$$

The average frequency of method *j*, call it f_j, is given by:

$$f_j = \frac{1}{N}\sum_i \frac{x_{ij}}{n_i}.$$

Next we sum the frequencies over all studies and do some rearranging:

$$\sum_j f_j = \sum_j \frac{1}{N}\sum_i \frac{x_{ij}}{n_i} = \frac{1}{N}\sum_i \sum_j \frac{x_{ij}}{n_i} = \frac{1}{N}\sum_i \left(\frac{1}{n_i}\sum_j x_{ij}\right) = \frac{1}{N}\sum_i T_i.$$

In the last step we see that summing all of the average frequencies is equivalent to averaging T_i (the mean number of tortures per individual in study *i*) over all studies. In other words, summing the average frequencies yields the average number of reported tortures per individual (across all studies).

© 2023 Milewski A et al. *JAMA Network Open.*

eTable 1. Categories for the Data Extraction Form

Article information
Article
Covidence number
First author
Title
Year published
Journal
Study design
Article's definition of torture
Does the study meet WMA criteria for torture?
Are mechanisms of torture specified?
Regions
Country hosting researchers
Location where researchers encountered population
Location of torture
Nationality of tortured individuals
Study population
Total number of tortured individuals*
Number of men
Number of women

Torture methods

TI	Name of method	Descriptors†	No. of individuals	Describe (if "other")
**PHYSICAL TORTURE**				
1	Beating or blunt-force trauma	Kicking, hitting with hands or dull object (e.g., baton), beating over the ears with cupped hands (telefono), bone breaking/crushing, trampling, stood on		
2	Electrical torture	Electrical shocks, electrocution, shocks to part of the body		
3	Burning	Combustion, burns with object, cigarette burns, burns with molten plastic		
4	Water torture	Drowning, dunking, submersion in water, submarino		
5	Pulling or dragging	Dragging by limbs or by hair, pulling on limbs or hair, dragging across the floor		
6	Asphyxiation or suffocation	Choking, placing bag over head, suffocation with plastic bag, hooding, restriction of breathing, suffocation with hands or object, head placed in a bag with acid/gasoline/petrol/chili, dry submarino, capucha, "la bolsa"		
7	Muscle crushing with roller	Ghotna, log roller, rolling of poles, ghotna between thighs with ankles tied together, ghotna in popliteal fossae and knees forcibly flexed, ghotna rolled down thighs/calves		
8	Sharp objects or penetrating trauma	Stabbed (e.g., with knife or nail), impaled		
9	Gunshot	Shot in part of the body but not killed		

© 2023 Milewski A et al. *JAMA Network Open.*

10	Suspension	Hanging, Palestinian hanging, suspension from rod, suspension with rope, poulet roti position, Dhar-machakra, the chicken, hanging from a stick between knees and arms bound tightly together, parrot perch
11	Foot whipping	Falanga, falaka, bastinado
12	Whipping	Flagellation, flogging
13	Starvation or dehydration	Deprivation of food or water
14	Deprivation of medical care	
15	Other forced positions	Carrying heavy loads/people over long distances, forced crouching, stretching legs beyond 180 degrees, abduction of hips, fixation in a car tire (dunlop method), used as a human shield, "scorpion position", forced gymnastics
16	Binding or restricted movement	Rope bondage, handcuffs, tying of hands, kandoya, chained to others, restriction of movement (e.g., placed in sack/box/very small space), incaprettamento, tied to an object
17	Forced prolonged standing	Forced standing with weight on
18	Stretching extremities	Stretched by limbs
19	Other physical torture	Subjected to physical torture without further specification; or, specifications did not match a category (e.g., amputation, removal of eyes, removal of appendages, forced to donate blood, human bite, tight rubber band placed around head, pinching, violent take-down)
20	Denailing	Needles under toes or fingernails
21	Removal of teeth	Extraction of teeth, grinding of teeth with a file
22	Stoning	
23	Pharmacological torture	Forced to take harmful or non-medicinal drugs, forced to take medications, administered anesthetic, injected with unknown chemical
24	Chemicals or irritants	Acid/gasoline/petroleum/chili powder/pepper powder in eyes, orifices, or on body; salt on wounds
**SENSORY TORTURE**		
25	Exposure to extreme hot/cold	Cold showers, climatic torture
26	Exposure to bright light	Forced solar gazing, exposed to heat/sun/strong light
27	Loud noises	Loud music, sound
28	Deprivation of light	Complete darkness
29	Other sensory torture	Subjected to sensory torture without further specification
**SEXUAL TORTURE**		
30	Rape	Including rape while being recorded, pregnancy from rape
31	Sexual assault or abuse	Fondling of genitals, forced to perform oral sex/fallatio, attempted rape
32	Sexual enslavement	
33	Genital trauma	Twisting, stretching, squeezing, beating of genitals; heavy objects hung from genitals
34	FGM/C	Female genital mutilation or cutting (all types)
35	Other sexual torture	Subjected to sexual torture without further specification; or, specifications did not match a category (e.g., sexual advances, forced incest, sex in exchange for gifts/food, forced to perform sexual acts with corpses)

© 2023 Milewski A et al. *JAMA Network Open.*

PSYCHOLOGICAL TORTURE	
36 Sham executions	Mock executions
37 Stripped naked	Forced nudity
38 Verbal abuse or threats	Threats of rape, threats of death, threats to family or other, threats of further torture, false accusations, threats of FGM/C, threats of sterility or castration
39 Fluctuating interrogator attitude	
40 Witnessing torture	Including physical, sensory, sexual and psychological torture
41 Other psychological torture	Subjected to psychological torture or humiliation without further specification; or, specifications did not match a category (e.g., forced defecation and vomiting, induced anal incontinence, forced ingestions or consumptions)
42 Isolation or solitary confinement	
43 Blindfolding	
44 Prevention of hygiene or exposure to infested surroundings	Prevention of urination/defecation, lack of toilet, denial of privacy, kept in an infested cell or prison without sanitation, prevention of bathing, contamination of food, dirty conditions leading to ill health, immersion in dirty fluid
45 Sleep deprivation	

TI - Torture Index, this index is referenced in **Figure 3**.
*This number includes only the individuals for whom a torture method was described. If two articles used the same sample or sub-sample of torture victims, then the article with more detail about the torture methods was included and the other article was excluded; if both articles included identical information about the torture inflicted, then the older article was included.
†These lists are not exhaustive; they capture examples of the types of descriptors encountered and the categories with which they corresponded.

© 2023 Milewski A et al. *JAMA Network Open.*

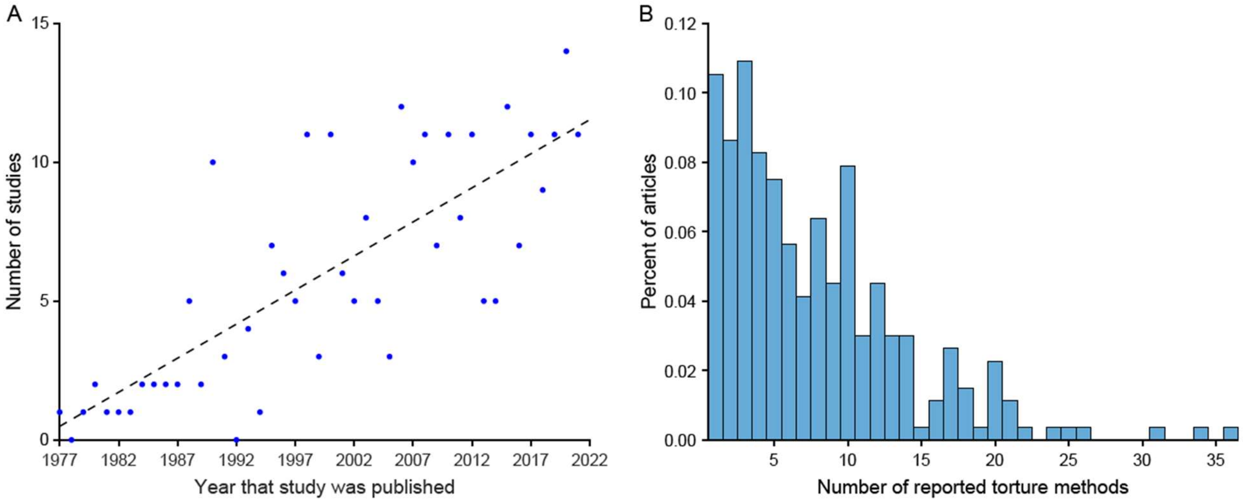

eFigure 2. Publication Years and Distribution of Reported Torture Methods for the Included Studies
(A) A scatter plot displays the number of studies that were published each year. The slope of the best-fit line (dashed, black line) is 0.25 articles per year, indicating that, on average, 0.25 more articles were published per year than the preceding year. Not shown is the single article published in 1947. (B) A bar graph portrays the distribution for the number of torture methods reported by the included articles.

© 2023 Milewski A et al. *JAMA Network Open.*

eTable 2: Characteristics of Included Studies

Study ID	Study design	Region(s) hosting researchers	Region(s) where researchers encountered population	Location of torture	Nationality of tortured individuals	Total tortured individuals (N)	Men (N)	Women (N)	Unique torture methods (N)
Aalund 1990	cross-sectional	Denmark	Chile	Chile	Chile	121	NS	NS	6
ABILDGAARD 1984	prospective blind-comparison-to-gold-standard study	Denmark	Greece	Greece	Greece	21	18	4	10
Adinkrah 2011	case-series	United States	Ghana	Ghana	Ghana	3	1	2	7
Agger 1990	case series	Denmark	Denmark	Africa, Middle East	Africa, Middle East	2	2	0	14
Aksaray 2000	cross-sectional	Turkey	Turkey	Turkey	Turkey	1	0	1	2
Alayarian 2009	serial cross-sectional	United Kingdom	United Kingdom	Africa	Africa	3	3	0	8
Allden 1998	case-series	United States	United States	Vietnam	Vietnam	1	0	1	1
Allodi 1982	cross-sectional	Canada	Canada	Latin America	Latin America	41	32	9	14
Allodi 1990	cross-sectional	Canada	Canada	Latin America	Latin America	56	28	28	18
Alpak 2015	cross-sectional	Turkey	Turkey	Syria	Syria	148	NS	NS	2
Alqassab 2021	case-report	Jordan, France	Jordan	Middle East	Middle East	1	1	0	1
Altun 2003	case-report	Turkey	Turkey	Turkey	Turkey	1	1	0	1
AmoneP'Olak 2009	cross-sectional, case-series	Netherlands	Uganda	Uganda	Uganda	14	8	6	10
Amris 2007	case-series	Denmark	United Kingdom	Saudi Arabia	United Kingdom	5	5	0	11
Amris 2009	case-control	Denmark, Turkey	Turkey	NS	NS	5	5	0	1
Aon 2018	qualitative	Denmark	Tanzania	Tanzania	Tanzania	14	12	2	17
Arge 2014	cross-sectional/case-series	Denmark	Denmark	Middle East, Former Soviet Republics, Africa, Asia	Middle East, Former Soviet Republics, Africa, Asia	33	29	4	3
Aron 1991	qualitative & case-report	United States	United States	El Salvador	El Salvador	1	0	1	2
Asgary 2006	cross-sectional	United States	United States	Bangladesh, India, Russian Federation,	Bangladesh, India, Russian Federation,	89	77	14	26

© 2023 Milewski A et al. *JAMA Network Open*.

Study ID	Study design	Region(s) hosting researchers	Region(s) where researchers encountered population	Location of torture	Nationality of tortured individuals	Total tortured individuals (N)	Men (N)	Women (N)	Unique torture methods (N)
				Pakistan, Montenegro, China, Guinea, Egypt, Sierra Leone, Togo, Liberia, Albania	Mauritania, Pakistan, Montenegro, China, Guinea, Egypt, Sierra Leone, Togo, Liberia, Albania				
Asgary 2013	cross-sectional	United States	United States	Guinea, Cameroon, Russian Federation, El Salvador, Congo, Chad, Mauritania, Ghana, Central African Republic, Gambia, Ethiopia, Belarus, Albania, India, Nepal, Guatemala, Colombia, Mexico	Guinea, Cameroon, Russian Federation, El Salvador, Congo, Chad, Mauritania, Ghana, Central African Republic, Gambia, Ethiopia, Belarus, Albania, India, Nepal, Guatemala, Colombia, Mexico	30	21	9	19
Asirdizer 2004	case-series	Turkey	Turkey	Turkey	Turkey	19	19	0	3
Badiee 2015	portraiture	United States	United States	Iran	Iran	1	0	1	4
Barber 2011	case report	Canada	Canada	Chile	Chile	1	1	0	1
BASOGLU 1992	case report	United Kingdom	United Kingdom	Middle East	Middle East	1	1	0	22
Basoglu 1997	case-control, cross-sectional	United Kingdom, United States, Turkey	Turkey	Turkey	Turkey	89	53	36	3
Basoglu 2004	case report	United Kingdom, Sweden	Sweden	Turkey	Turkey	1	1	0	31
Berger 1980	Case series	Canada	Canada	Uruguay, Chile, El Salvador, South Africa	Uruguay, Chile, El Salvador, South Africa	8	7	1	16
Berman 2006	qualitative study (cross-sectional)	Canada	Canada	Bosnia and Herzegovina, Guatemala, Chile	Bosnia and Herzegovina, Guatemala, Chile	3	0	3	5

© 2023 Milewski A et al. *JAMA Network Open*.

Study ID	Study design	Region(s) hosting researchers	Region(s) where researchers encountered population	Location of torture	Nationality of tortured individuals	Total tortured individuals (N)	Men (N)	Women (N)	Unique torture methods (N)
Bichescu 2005	case-control	Germany, Romania, Italy	Romania	Romania	Romania	59	58	1	14
Bloom 1995	case-series	Israel	Israel	NS	NS	2	2	0	3
Boersma 2003	cross-sectional	United States	United States	Africa, Europe, Central America, Middle East, South America, Southeastern Asia	Africa, Europe, Central America, Middle East, South America, Southeastern Asia	16	7	9	3
Boos 2000	cross-sectional	United Kingdom, Germany	Germany	Germany	Germany	81	66	15	5
Bork 1997	case-report	Germany	Germany	Kuwait	Kuwait	1	1	0	1
Bouwer 1998	cross-sectional	South Africa, New Zealand	South Africa	South Africa	South Africa	8	8	0	6
Bradley 2006	cross-sectional	United Kingdom	United Kingdom	Turkey	Turkey	97	83	14	10
BroddaJansen 2020	case-series	Sweden	Sweden, Denmark	Iraq	Iraq	1	0	1	3
Busch 2015	cross-sectional	Denmark	Denmark	Middle East, Asia, Europe, Africa	Middle East, Asia, Europe, Africa	154	146	8	10
Byard 2012	case-report	Australia, Malaysia	Malaysia	Malaysia	Malaysia	1	1	0	2
CALLAGHAN 1993	case-report	United Kingdom	United Kingdom	Africa	Africa	1	0	1	3
Carlsson 2006	cross-sectional	Denmark	Denmark	Iraq, Afghanistan, Iran, Other	Iraq, Afghanistan, Iran, Other	63	63	0	9
Cathcart 1979	cross-sectional	Canada	Canada	Chile	Chile	11	8	3	13
Charlier 2018	case-report	France, Lebanon	France	Côte d'Ivoire	Côte d'Ivoire	1	1	0	2
Chaudhry 2008	cross-sectional	Pakistan	Pakistan	Pakistan	Pakistan	1820	1666	154	7
Choi 2017	cross-sectional	South Korea	South Korea	South Korea	South Korea	206	170	36	20
Chu 2013	cross-sectional	United States	United States	Africa, Asia, Eastern Europe, Other	Africa, Asia, Eastern Europe, Other	402	259	143	5

© 2023 Milewski A et al. *JAMA Network Open*.

Study ID	Study design	Region(s) hosting researchers	Region(s) where researchers encountered population	Location of torture	Nationality of tortured individuals	Total tortured individuals (N)	Men (N)	Women (N)	Unique torture methods (N)
Clement 2017	cross-sectional	France	France	Guinea-Bissau, Congo DRC, Guinea, Russian Federation, Nigeria, Serbia, Angola, Sudan, Chad, Eritrea, Central Africa, Georgia, Ethiopia, Rwanda, Sri Lanka, Cameroon, Mongolia, Afghanistan, China, Kazakhstan, Somalia, Turkey, Mauritania, Serbia, Sierra Leone, Benin, Burkina Faso, Djibouti, Ecuador, Gabon, Mauritius, India, Iraq, Kurdistan, Tunisia, Madagascar, Morocco, Republic of Caucasus, Senegal, Syria, Ukraine	Guinea-Bissau, Congo DRC, Guinea, Russian Federation, Nigeria, Serbia, Angola, Sudan, Chad, Eritrea, Central Africa, Georgia, Ethiopia, Rwanda, Sri Lanka, Cameroon, Mongolia, Afghanistan, China, Kazakhstan, Somalia, Turkey, Mauritania, Serbia, Sierra Leone, Benin, Burkina Faso, Djibouti, Ecuador, Gabon, Mauritius, India, Iraq, Kurdistan, Tunisia, Madagascar, Morocco, Republic of Caucasus, Senegal, Syria, Ukraine	570	391	179	6
Cohen 2017	cross-sectional	United Kingdom	United Kingdom	Sri Lanka	Sri Lanka	68	61	7	10
Cooper 2011	case-report	United Kingdom	United Kingdom	Africa	Africa	1	1	0	4
Crescenzi 2002	case-control	India, Netherlands	India	China	China	150	94	56	18
Crosby 2010	case-series	United States	United States	Middle East, Central Africa, Eastern Africa	Middle East, Central Africa, Eastern Africa	5	3	2	6
Dalgaard 2021	cross-sectional	Denmark	Denmark	Iraq, Iran, Lebanon, Chile, Palestinian Territory, Turkey, Lebanon, Kurdistan, Other	Iraq, Iran, Lebanon, Chile, Palestinian Territory, Turkey, Lebanon, Kurdistan, Other	1135	978	157	11
DandeniyaArachchi 2019	case-report	Sri Lanka	Sri Lanka	Sri Lanka	Sri Lanka	1	1	0	11

© 2023 Milewski A et al. *JAMA Network Open.*

Study ID	Study design	Region(s) hosting researchers	Region(s) where researchers encountered population	Location of torture	Nationality of tortured individuals	Total tortured individuals (N)	Men (N)	Women (N)	Unique torture methods (N)
Danielsen 1981	case-series	Denmark, Canada	Denmark, Canada	South America, Iraq, India	South America, Iraq, India	3	2	1	7
Danneskiold-Samsoe 2006	longitudinal	Denmark	Denmark	Middle East, Europe, Latin America, Africa	Middle East, Europe, Latin America, Africa	21	21	0	10
Daugaard 1983	cross-sectional	Denmark	Denmark	Greece	Greece	10	10	0	2
de Zoysa 2007	cross-sectional	Sri Lanka	Sri Lanka	Sri Lanka	Sri Lanka	90	83	7	4
deFouchier 2012	cross-sectional	France, United Kingdom	France, United Kingdom	Sub-Saharan Africa	Sub-Saharan Africa	52	29	23	2
deJong 2008	cross-sectional	Netherlands, Canada	Indian-held Kashmir	India	India	66	44	22	24
Deol 2018	cross-sectional	India	India	Kashmir	Kashmir	25	NS	NS	10
Deps 2021	cross-sectional	France	France	Guinea, Côte d'Ivoire, Congo DRC, Sub-Saharan Africa, Northern Africa, Sudan, Middle East	Guinea, Côte d'Ivoire, Congo DRC, Sub-Saharan Africa, Northern Africa, Sudan, Middle East	9	NS	NS	10
Deps 2021	cross-sectional	France	France	Sudan, Guinea, Morocco, Côte d'Ivoire, Western Sahara, Somalia, Northern Africa, Western Africa	Sudan, Guinea, Morocco, Côte d'Ivoire, Western Sahara, Somalia, Northern Africa, Western Africa	99	NS	NS	3
Dibaj 2017	Case series	Norway	Norway	Middle East, Caucasus, Central Africa	Middle East, Caucasus, Central Africa	6	5	1	20
Dickson-Lowe 2021	cross-sectional	United Kingdom, South Africa	South Africa	South Africa	South Africa	138	132	6	2
Dolma 2006	cross-sectional	Canada, United States, United Kingdom	Nepal	China	China	20	NS	NS	8
Domovitch 1984	cross-sectional	Canada, United States	Canada	Chile	Chile	104	91	13	21

© 2023 Milewski A et al. *JAMA Network Open.*

Study ID	Study design	Region(s) hosting researchers	Region(s) where researchers encountered population	Location of torture	Nationality of tortured individuals	Total tortured individuals (N)	Men (N)	Women (N)	Unique torture methods (N)
East 2018	cross-sectional	United States	United States	Somalia	Somalia	12	0	12	4
Edston 2007	cross-sectional/case-series	Sweden	Sweden	Bangladesh, Iran, Uganda, Congo DRC, Peru, Syria, Turkey, Azerbaijan, India, Somalia, Angola, Bosnia and Herzegovina, Colombia, Cuba, Ecuador, Ethiopia, Kenya, Serbia, Montenegro, Nigeria, Pakistan, Russian Federation	Bangladesh, Iran, Uganda, Congo DRC, Peru, Syria, Turkey, Azerbaijan, India, Somalia, Angola, Bosnia and Herzegovina, Colombia, Cuba, Ecuador, Ethiopia, Kenya, Serbia, Montenegro, Nigeria, Pakistan, Russian Federation	63	0	63	13
Einolf 2018	cross-sectional	United States	Iraq	Iraq	Iraq	47	20	27	17
ElSarraj 1996	cross-sectional	Palestinian Territory, Finland, United Kingdom	Palestinian Territory	Israel	Palestinian Territory	550	550	0	11
Engstrom 2004	case-series	United States	United States	Eastern Africa, Central America	Eastern Africa	2	1	1	8
FAHY 1988	case report	United Kingdom	United Kingdom	Africa	Africa	1	0	1	3
Fernandes 2019	case-series	Australia	Australia	Sri Lanka	Sri Lanka	2	1	1	10
Fernandez 2001	case-report	Sweden, United States	Sweden	NS	NS	1	1	0	8
Ferrada-Noli 1998	cross-sectional	Sweden, United States	Sweden	Middle East, Africa, Yugoslavia, Eastern Europe, Latin America	Middle East, Africa, Yugoslavia, Eastern Europe, Latin America	27	NS	NS	9
Fetherston 2020	case-series/cross-sectional	United Kingdom	United Kingdom	Congo DRC, Sudan	Congo DRC, Sudan	17	7	10	2

© 2023 Milewski A et al. *JAMA Network Open*.

Study ID	Study design	Region(s) hosting researchers	Region(s) where researchers encountered population	Location of torture	Nationality of tortured individuals	Total tortured individuals (N)	Men (N)	Women (N)	Unique torture methods (N)
Fidaner 1991	cross-sectional	Turkey	Turkey	NS	NS	33	22	11	9
Fischman 1990	case-series	United States	United States	Central America, South America	Central America, South America	2	1	1	5
Fornazzari 1990	cross-sectional	Canada	Canada	Chile, Guatemala, Uruguay, El Salvador, Argentina, Bolivia	Chile, Guatemala, Uruguay, El Salvador, Argentina, Bolivia	36	0	36	3
Forrest 1995	cross-sectional	United Kingdom	United Kingdom	India	India	31	31	0	6
Gavagan 1997	case-series	United States	United States	Guatemala	Guatemala	3	2	1	4
Ghaddar 2016	cross-sectional	Lebanon	Lebanon	Lebanon	Lebanon	67	0	67	10
Ghaleb 2014	cross-sectional	Egypt	Egypt	Egypt	Egypt	367	336	31	7
Gniadecka 1995	case-report	Denmark	Denmark	NS	NS	1	1	0	1
Gonsalves 1990	cross-sectional qualitative	United States	United States	Chile	Chile	20	16	4	6
Gorst-Unsworth 1993	cross-sectional	United Kingdom	United Kingdom	Iran, Iraq, Somalia, Turkey, South Africa, Angola, Ethiopia, Guinea-Bissau, Suriname, Uganda, United Kingdom, Congo DRC	Iran, Iraq, Somalia, Turkey, South Africa, Angola, Ethiopia, Guinea-Bissau, Suriname, Uganda, United Kingdom, Congo DRC	31	27	4	20
Gorst-Unsworth 1998	cross-sectional	United Kingdom	United Kingdom	Iraq	Iraq	84	84	0	3
Gray 2001	case report	United States	United States	Africa	Africa	1	0	1	5
Green 2007	case-series	United States	United States	Afghanistan	Afghanistan	1	1	0	1
Gregurek 2001	cross-sectional	Croatia	Croatia	Croatia	Croatia	1	1	0	2
Grodin 2008	case-series	United States	United States	China, Lebanon, Belarus, Congo DRC	China, Lebanon, Belarus, Congo DRC	4	3	1	6
Grossman 1996	cross-sectional	United States	United States	Japan	United States	75	75	0	2

© 2023 Milewski A et al. *JAMA Network Open*.

Study ID	Study design	Region(s) hosting researchers	Region(s) where researchers encountered population	Location of torture	Nationality of tortured individuals	Total tortured individuals (N)	Men (N)	Women (N)	Unique torture methods (N)
Gulden 2010	cross-sectional	United States	United States	Ethiopia	Ethiopia	512	282	230	36
Guzel 2015	case-report	Turkey	Turkey	Syria	Syria	1	1	0	1
Haar 2019	cross-sectional qualitative	United States	Bangladesh	Myanmar	Myanmar	114	78	36	4
Halvorsen 2010	longitudinal uncontrolled trial	Norway	Norway	Iraq, Afghanistan, Eritrea, Ethiopia, Iran, Serbia, Sudan, Togo	Iraq, Afghanistan, Eritrea, Ethiopia, Iran, Serbia, Sudan, Togo	16	13	3	14
Harris 2019	case-series	United States	United States	Eastern Africa, Northern Africa	Eastern Africa, Northern Africa	2	1	1	5
Hartmann 2009	case-report	Germany	Germany	Algeria	Algeria	1	1	0	8
Hexom 2012	cross-sectional	United States	United States	United States, Colombia, Dominican Republic, China, Croatia, El Salvador, Honduras, Morocco, Philippines, Sierra Leone	United States, Colombia, Dominican Republic, China, Croatia, El Salvador, Honduras, Morocco, Philippines, Sierra Leone	29	20	9	3
Highfield 2012	case-series/cross-sectional	United States	United States	Somalia, China, Uganda	Somalia, China, Uganda	3	1	2	4
Holtz 1998	case-control	United States	India	China	China	35	7	28	21
Hondius 2000	cross-sectional	Netherlands	Netherlands	Latin America, Western Asia, Turkey, Iran	Latin America, Western Asia, Turkey, Iran	328	NS	NS	13
Hooberman 2010	cross-sectional	United States	United States	Africa, Asia, Europe, South America	Africa, Asia, Europe, South America	75	44	31	3
Hougen 1988	cross-sectional	Denmark	Denmark	Lebanon	Lebanon	12	12	0	13
Hougen 1988	cross-sectional	Denmark	Denmark	Turkey	Turkey	14	12	2	10
Hughes 2012	qualitative	United Kingdom	Sri Lanka	Sri Lanka	Sri Lanka	5	5	0	11
Hunt 2008	cross-sectional	United States	United States	Europe, Pacific Islands, Korea, Vietnam	United States	265	265	0	5

© 2023 Milewski A et al. *JAMA Network Open*.

Study ID	Study design	Region(s) hosting researchers	Region(s) where researchers encountered population	Location of torture	Nationality of tortured individuals	Total tortured individuals (N)	Men (N)	Women (N)	Unique torture methods (N)
Iacopino 2001	cross-sectional	United States	North Macedonia, Albania	Serbia	Albania	506	NS	NS	6
Iacopino 2011	case-series	United States	Guantanamo, United States	Cuba-Guantánamo Bay	NS	9	NS	NS	12
Ibrahim 2017	cross-sectional	Germany, Iraq	Iraq	Iraq	Syria	91	51	40	8
Ilenia 2021	cross-sectional	Italy	Italy	Africa, Bangladesh, Pakistan, Libya	Nigeria, Guinea, Gambia, Senegal, Mali, Côte d'Ivoire, Ghana, Guinea-Bissau, Liberia, Burkina Faso, Sierra Leone, Eastern Africa, Somalia, Eritrea, Pakistan, Bangladesh, Afghanistan, Europe, Middle East	114	NS	NS	15
Jayawickreme 2019	cross-sectional	United States, United Kingdom	Sri Lanka	Sri Lanka	Sri Lanka	98	NS	27	5
Jayawickreme 2020	cross-sectional	United States, Australia	Sri Lanka	Sri Lanka	Sri Lanka	123	NS	NS	5
Jensen 2013	case report	Denmark	Denmark	Iraq	Iraq	1	1	0	10
Jorgensen 2015	serial cross-sectional	Denmark, India	India	India	India	470	253	217	3
Kagee 2004	qualitative	South Africa	South Africa	South Africa	South Africa	1	NS	NS	3
Kanninen 2000	case-report	Finland	Palestinian Territory	Israel	Palestinian Territory	31	31	0	4
Kastrup 1986	cross-sectional	Denmark	Denmark	NS	NS	38	NS	NS	14
Kaur 2020	cross-sectional/case-series	United States	United States	NS	NS	20	9	11	10

© 2023 Milewski A et al. *JAMA Network Open*.

Study ID	Study design	Region(s) hosting researchers	Region(s) where researchers encountered population	Location of torture	Nationality of tortured individuals	Total tortured individuals (N)	Men (N)	Women (N)	Unique torture methods (N)
Keatley 2015	cross-sectional	Canada, United States	United States	Western Africa, Central Africa, Central Asia, Eastern Asia, Eastern Europe, Other	Western Africa, Central Africa, Central Asia, Eastern Asia, Eastern Europe, Other	85	59	26	1
Keller 1998	case-report	United States	United States	Africa, China	Africa, China	3	2	1	14
Keller 2002	case series	United States	United States	NS	NS	1	0	1	8
Keller 2006	case series	United States	Jordan	Iraq	Iraq	2	2	0	3
Keller 2006	cross-sectional	United States	United States	Western Africa, Central Africa, Africa, Eastern Asia, Asia, Eastern Europe, China, Sierra Leone, Guinea	Western Africa, Central Africa, Africa, Eastern Asia, Asia, Eastern Europe, China, Sierra Leone, Guinea	325	199	126	3
Keller 2014	case report (I didn't consider the descriptive stats in the beginning which would be cross-sectional because part of those data mentioned were cited from previous articles)	United States	United States	Africa	Africa	1	0	1	10
Keten 2020	cross-sectional	Germany	NA†	Turkey	Turkey	17	NS	NS	5
Kienzler 2019	qualitative cross-sectional	United Kingdom, Canada	India	India	India	1	1	0	3
Kinyanda 2010	cross-sectional	Uganda	Uganda	Uganda	Uganda	180	16	164	3
Kinzie 1987	case series	United States	United States	Cambodia, Iran	Cambodia, Iran	2	0	2	5
Kinzie 2012	uncontrolled, non-random,	United States	United States	Iran, Afghanistan, Ethiopia, Somalia	Iran, Afghanistan, Ethiopia, Somalia	22	9	13	4

© 2023 Milewski A et al. *JAMA Network Open.*

Study ID	Study design	Region(s) hosting researchers	Region(s) where researchers encountered population	Location of torture	Nationality of tortured individuals	Total tortured individuals (N)	Men (N)	Women (N)	Unique torture methods (N)
	prospective clinical study								
Kizilhan 2018	cross-sectional	Germany, Iraq	Germany	Iraq, Syria	Iraq	296	0	296	7
Knipscheer 2015	cross-sectional	Netherlands	Netherlands	Afghanistan, Iraq, Egypt, Libya, Middle East, Burkina Faso, Eritrea, Congo DRC, Sub-Saharan Africa, Bosnia and Herzegovina, Yugoslavia, Croatia, Balkans	Afghanistan, Iraq, Egypt, Libya, Middle East, Burkina Faso, Eritrea, Congo DRC, Sub-Saharan Africa, Bosnia and Herzegovina, Yugoslavia, Croatia, Balkans	495	NS	NS	1
Kozaric-Kovacic 1995	cross-sectional and case-series	Croatia	Croatia	Bosnia and Herzegovina, Croatia	Bosnia and Herzegovina, Croatia	25	0	25	5
Kucukalic 2003	uncontrolled, non-random, prospective clinical study	Bosnia and Herzegovina	Bosnia and Herzegovina	Bosnia and Herzegovina, Croatia, Other	Bosnia and Herzegovina, Croatia, Other	108	74	34	2
Larsen 1987	case series	Denmark, Philippines	Philippines	Philippines	Philippines	3	0	3	16
Lawson 1999	cross-sectional	United Kingdom	United Kingdom	Sierra Leone	Sierra Leone	36	10	26	6
Leaman 2012	cross-sectional	United States	United States	Cameroon, Middle Africa, Ethiopia, Eastern Africa, Kenya, Sierra Leone, Togo, Burundi, Mali	Cameroon, Middle Africa, Ethiopia, Eastern Africa, Kenya, Sierra Leone, Togo, Burundi, Mali	131	56	75	25
Lerner 2016	cross-sectional	United States, Canada	United States	Western Africa, Asia, Africa, Eastern Europe, Central Africa	Western Africa, Asia, Africa, Eastern Europe, Central Africa	267	179	88	10
Leth 2005	cross-sectional	Denmark	Denmark	Iran, Syria, Iraq, Lebanon, Palestinian Territory, Sri Lanka, India, Afghanistan, Pakistan, Cameroon,	Iran, Syria, Iraq, Lebanon, Palestinian Territory, Sri Lanka, India, Afghanistan, Pakistan, Cameroon,	59	59	0	18

© 2023 Milewski A et al. *JAMA Network Open.*

Study ID	Study design	Region(s) hosting researchers	Region(s) where researchers encountered population	Location of torture	Nationality of tortured individuals	Total tortured individuals (N)	Men (N)	Women (N)	Unique torture methods (N)
				Burundi, Rwanda, Sudan, Libya, Yugoslavia	Burundi, Rwanda, Sudan, Libya, Yugoslavia				
Ley 2018	case-report	Austria, Spain	Austria	Southern Asia	Southern Asia	1	1	0	1
Lie 2002	serial cross-sectional	Norway	Norway	Bosnia and Herzegovina, Serbia, Middle East, Asia, Africa, Latin America	Bosnia and Herzegovina, Serbia, Middle East, Asia, Africa, Latin America	84	NS	NS	4
Loncar 2006	cross-sectional	Croatia	Croatia	Croatia, Bosnia and Herzegovina	Croatia, Bosnia and Herzegovina	68	0	68	9
Loncar 2010	cross-sectional	Croatia	Croatia	Croatia, Bosnia and Herzegovina	Croatia, Bosnia and Herzegovina	60	60	0	7
Longstreth 2021	case series	United States	United States	Cameroon, Sierra Leone	Cameroon, Sierra Leone	10	8	2	1
Loutan 1999	cross-sectional	Switzerland	Switzerland	Africa, Europe, Asia, Latin America, Other	Africa, Europe, Asia, Latin America, Other	104	98	6	2
Lunde 1990	cross-sectional	Denmark	Denmark	Europe, Latin America, Africa, Indonesia, Turkey, Middle East, Far East, Africa, Europe	Europe, Latin America, Africa, Indonesia, Turkey, Middle East, Far East, Africa, Europe	100	NS	NS	1
Lykke 2002	cross-sectional	Nepal	Nepal	Nepal	Nepal	63	58	5	17
Magaloni 2020	cross-sectional	United States	NA§	Mexico	Mexico	75573	NS	NS	9
Magli 2019	cross-sectional	Italy	Italy	Africa, Asia, Nigeria, Côte d'Ivoire, Somalia, Gambia, Mali, Senegal, Togo, Algeria, Pakistan, Afghanistan, Iran	Nigeria, Côte d'Ivoire, Somalia, Gambia, Mali, Senegal, Togo, Algeria, Pakistan, Afghanistan, Iran	225	133	92	3
Malik 1993	case-series	India	India	India	India	10	10	0	3
MALIK 1995	case-series	India	India	India	India	34	34	0	3
Mannan 2011	case-series	Pakistan	Pakistan	Pakistan	Pakistan	1	1	0	1
Martell 2020	cross-sectional	United States	United States	Burkina Faso, Guinea, Congo DRC, Cameroon,	Burkina Faso, Guinea, Congo DRC, Cameroon,	24	0	24	1

© 2023 Milewski A et al. *JAMA Network Open.*

Study ID	Study design	Region(s) hosting researchers	Region(s) where researchers encountered population	Location of torture	Nationality of tortured individuals	Total tortured individuals (N)	Men (N)	Women (N)	Unique torture methods (N)
				Mali, Côte d'Ivoire, Chad, Nigeria, Central African Republic, Sudan, Togo, Other	Mali, Côte d'Ivoire, Chad, Nigeria, Central African Republic, Sudan, Togo, Other				
Masmas 2008	serial cross-sectional?	Denmark	Denmark	Afghanistan, Iraq, Iran, Russian Federation, Syria, Other	Afghanistan, Iraq, Iran, Russian Federation, Syria, Other	64	55	9	10
Matos 2021	cross-sectional & qualitative	Portugal, United States	Portugal	Syria	Syria	6	6	0	8
McColl 2010	serial cross-sectional	Denmark, South Africa, Egypt, Mexico, Honduras, Palestinian Territory	Egypt, Honduras, Mexico, Palestinian Territory, South Africa	Egypt, Honduras, Mexico, Palestinian Territory, South Africa, Africa	Egypt, Honduras, Mexico, Palestinian Territory, South Africa, Africa	306	NS	NS	6
McKenzie 2017	case-series	United States	United States	Congo DRC, Western Africa, Africa	Congo DRC, Western Africa, Africa	3	2	1	8
MemberCentersoftheNationalConsortiumofTortureTreatmentPrograms(NCTTP) 2015	cross-sectional (subset: serial cross-sectional)	United States	United States	Africa, Asia, Latin America, Europe, Pacific Islands	Africa, Asia, Latin America, Europe, Pacific Islands	3096	1744	1352	11
Miller 1989	cross-sectional	United States	United States	Japan, Germany	NS	44	44	0	5
Mirzaei 1998	case-report	Austria	Austria	NS	NS	25	22	3	2
Moisander 2003	cross-sectional	Sweden	Sweden	Bangladesh, Iran, Peru, Syria, Turkey, Uganda	Bangladesh, Iran, Peru, Syria, Turkey, Uganda	160	139	21	12
Mollica 1998	cross-sectional	United States	United States	Vietnam	Vietnam	73	73	0	4
Mollica 2007	serial cross-sectional	United States	Croatia	Bosnia and Herzegovina	Bosnia and Herzegovina	104	NS	NS	20

© 2023 Milewski A et al. *JAMA Network Open*.

Study ID	Study design	Region(s) hosting researchers	Region(s) where researchers encountered population	Location of torture	Nationality of tortured individuals	Total tortured individuals (N)	Men (N)	Women (N)	Unique torture methods (N)
Mollica 2014	cross-sectional	United States	United States	Vietnam	Vietnam	68	68	0	5
Montgomery 1998	cross-sectional	Denmark	Denmark	Middle East	Middle East	22	17	5	9
Montgomery 2004	qualitative	Denmark	Denmark	Middle East	Middle East	2	1	1	2
Moreno 2000	case-report	United States	United States	Cameroon	Cameroon	1	0	1	7
Moreno 2003	cross-sectional	United States	NA‡	Mexico	Mexico	32	3	29	4
Moreno 2006	case-series	United States	United States	Africa, Latin America, Caribbean, Asia, Europe	Africa, Latin America, Caribbean, Asia, Europe	109	NS	NS	12
Moreno 2008	cross-sectional	United States	NA‡	Mexico	Mexico	39	36	3	12
Moreno 2015	cross-sectional	United States	Kyrgyzstan	Kyrgyzstan	Kyrgyzstan	10	10	0	17
Moreno 2020	cross-sectional	United States, Denmark, Taiwan	NA‡	Thailand	Thailand	49	49	0	12
Morentin 1995	cross-sectional	Spain	Spain	Spain	Spain	87	70	17	17
Morentin 1997	cross-sectional	Spain	Spain	Spain	Spain	99	NS	NS	8
Morentin 2008	cross-sectional	Spain	Spain	Spain	Spain	199	112	87	8
Munczek 1998	case report	United States	Honduras	Honduras	Honduras	1	1	0	9
Musisi 2000	cross-sectional	Uganda	Uganda	Uganda	Uganda	310	117	183	7
Neufeld 2021	case-series	United States	United States	NS	NS	2	2	0	2
Nguyen 2019	case report	United States	United States	Nigeria	Nigeria	1	1	0	10
Nickerson 2016	cross-sectional	Australia, United States	Australia	Iran, Afghanistan, Sri Lanka, Iraq, Serbia, Bhutan, Nigeria, Other	Iran, Afghanistan, Sri Lanka, Iraq, Serbia, Bhutan, Nigeria, Other	33	26	7	6

© 2023 Milewski A et al. *JAMA Network Open*.

Study ID	Study design	Region(s) hosting researchers	Region(s) where researchers encountered population	Location of torture	Nationality of tortured individuals	Total tortured individuals (N)	Men (N)	Women (N)	Unique torture methods (N)
Nieves-Grafals 2001	case series?	United States	United States	Central America	Central America	1	1	0	9
Nordin 2019	longitudinal uncontrolled unblinded non-randomized clinical study	Denmark, Sweden	Denmark	Iraq, Iran, Lebanon, Bosnia and Herzegovina, Afghanistan, Somalia, Syria, Egypt, Russian Federation, Turkey	Iraq, Iran, Lebanon, Bosnia and Herzegovina, Afghanistan, Somalia, Syria, Egypt, Russian Federation, Turkey	276	170	106	4
Norredam 2005	case-series	United States, Denmark	United States	Central Africa, Eastern Africa, Western Africa	Central Africa, Eastern Africa, Western Africa	3	3	0	4
Nou 2015	qualitative	United States	Cambodia	Cambodia	Cambodia	3	0	3	9
Olsen 2006	cross-sectional	Denmark	Denmark	Lebanon, Other	Iran, Iraq, Lebanon, Turkey, Other, Palestinian Territory	221	193	28	7
Olsen 2006	serial cross-sectional	Denmark	Denmark	Iraq, Afghanistan, Other	Iraq, Afghanistan, Other	69	61	8	8
Olsen 2007	serial cross-sectional	Denmark	Denmark	Iran, Iraq, Lebanon, Palestinian Territory, Other	Iran, Iraq, Lebanon, Palestinian Territory, Kurdistan, Other	139	126	13	12
Owens 2021	case-series	United Kingdom, South Africa	Spain	Spain	Spain	23	23	0	3
Ozkalipci 2013	cross-sectional	Denmark, Turkey	Turkey	Turkey	Turkey	97	80	17	1
Peel 1996	cross-sectional	United Kingdom	United Kingdom	Congo DRC	Congo DRC	81	68	13	10
Peel 2000	cross-sectional	United Kingdom	United Kingdom	Sri Lanka	Sri Lanka	38	38	0	7
Peel 2003	case-series	United Kingdom	United Kingdom	Western Africa, South Africa	Western Africa, South Africa	4	4	0	3
Peltzer 1999	qualitative	South Africa	South Africa	Uganda, Malawi	Uganda, Malawi	2	1	1	9
Perera 2007	cross-sectional	Sri Lanka	Sri Lanka	Sri Lanka	Sri Lanka	100	89	11	21

© 2023 Milewski A et al. *JAMA Network Open*.

Study ID	Study design	Region(s) hosting researchers	Region(s) where researchers encountered population	Location of torture	Nationality of tortured individuals	Total tortured individuals (N)	Men (N)	Women (N)	Unique torture methods (N)
Perez-Sales 2010	case-report	Spain	Spain	Spain	Spain	1	1	0	2
Perez-Sales 2016	cross-sectional, qualitative	Spain	Spain	Spain	Spain	45	NS	NS	12
Petersen 1985	case-control	Denmark	Spain	Spain	Spain	10	7	3	11
Petersen 1985	serial cross-sectional	Denmark	Greece	Greece	Greece	22	18	4	9
Petersen 1994	case series	Denmark	Indian-held Kashmir	India	India	7	7	0	12
Petersen 1995	case series	Denmark	Pakistan-held Kashmir	Pakistan	Pakistan	9	9	0	8
Petersen 2000	cross-sectional	Denmark	Thailand	Myanmar	Myanmar	19	NS	NS	4
Petersen 2019	cross-sectional	Denmark, Spain	Spain	Spain	Spain	202	160	42	10
Piwowarczyk 2017	cross-sectional	United States	United States	Uganda, Africa, Asia, Central America, Europe, Middle East, South America	Uganda, Africa, Asia, Central America, Europe, Middle East, South America	42	NS	NS	6
Pohlman 2017	case-report	Australia	Netherlands	Indonesia	Indonesia	2	1	1	7
Polat 2010	case-report	United States	United States	Cameroon	Western Africa	1	1	0	3
Pollanen 2002	case-report	Canada	Canada	NS	NS	1	1	0	2
Pollanen 2003	case-report	Canada, Timor-Leste	Timor-Leste	Timor-Leste	Timor-Leste	1	1	0	3
Pollanen 2016	case-report	Canada	NS	NS	NS	1	1	0	4
Priebe 1997	case-control	Germany	Germany	Iran	Iran	34	18	16	13
Prip 2008	case-control	Denmark	Denmark	Iraq, Iran	Iraq, Iran	11	11	0	1
Prip 2011	cross-sectional	Denmark	Denmark	Iraq, Iran, Africa, Asia, Latin America	Iraq, Iran, Africa, Asia, Latin America	71	63	8	1
Prip 2012	case-control	Denmark	Denmark	Middle East	Middle East	15	15	0	1
PUNAMAKI 1988	cross-sectional, qualitative	Finland	Palestinian Territory	Palestinian Territory	Palestinian Territory	40	NS	NS	14

© 2023 Milewski A et al. *JAMA Network Open.*

Study ID	Study design	Region(s) hosting researchers	Region(s) where researchers encountered population	Location of torture	Nationality of tortured individuals	Total tortured individuals (N)	Men (N)	Women (N)	Unique torture methods (N)
Ramsay 1993	cross-sectional	United Kingdom	United Kingdom	Africa, Middle East, Sri Lanka, South America, Europe	Africa, Middle East, Sri Lanka, South America, Europe	100	78	22	12
RanilSanjeewa 2017	case-report	Sri Lanka	Sri Lanka	Sri Lanka	Sri Lanka	1	1	0	2
Rashid 2012	case-series, qualitative, cross-sectional	India	India	India	India	5	4	1	11
Rasmussen 1980	cross-sectional	Denmark	Denmark, Greece, Spain, Ireland, Italy	Chile, Greece, Spain, Argentina, Ireland, Other	Chile, Greece, Spain, Argentina, Ireland, Other	135	109	26	4
RASMUSSEN 1990	cross-sectional	Denmark	Austria, Chile, Denmark, France, Germany, Greece, India, Italy, Middle East, Ireland, Norway, Spain, Switzerland	Argentina, Bolivia, Chile, Eritrea, Ethiopia, Greece, India, Indonesia, Iraq, Morocco, Ireland, Somalia, Spain, Syria, Tanzania, Uruguay	Argentina, Bolivia, Chile, Denmark, Eritrea, Ethiopia, Greece, India, Indonesia, Iraq, Ireland, Somalia, Spain, Switzerland, Tanzania, Uganda, Uruguay	200	161	39	10
Rasmussen 2007	cross-sectional	United States	United States	Guinea, Sierra Leone, Mauritania, Cameroon, Congo DRC, Togo, Liberia, Other	Guinea, Sierra Leone, Mauritania, Cameroon, Congo DRC, Togo, Liberia, Other	343	NS	NS	17
Rasmussen 2007	cross-sectional	United States, Canada	United States	India	India	61	44	17	17
Rasmussen 2011	cross-sectional	United States	United States	Western Africa, Eastern Asia, Central Africa, Southern Asia, Eastern Europe, Other	Western Africa, Eastern Asia, Central Africa, Southern Asia, Eastern Europe, Other	132	NS	NS	6
Ray 2006	case-control	Germany, United States	Germany	Turkey, Yugoslavia, Algeria	Turkey, Yugoslavia, Algeria	23	11	12	4
Reid 1988	qualitative	Australia	Australia	Vietnam, Iran, Afghanistan, Laos, Other	Vietnam, Iran, Afghanistan, Laos, Other	5	4	1	16

© 2023 Milewski A et al. *JAMA Network Open.*

Study ID	Study design	Region(s) hosting researchers	Region(s) where researchers encountered population	Location of torture	Nationality of tortured individuals	Total tortured individuals (N)	Men (N)	Women (N)	Unique torture methods (N)
Reid 1990	cross-sectional, case-series	Australia	Australia	Middle East, Cambodia	Middle East, Cambodia	2	1	1	12
Rivera-Holguin 2019	qualitative	Peru, Spain	Peru	Peru	Peru	5	NS	NS	4
Rodolico 2020	cross-sectional	Italy	Italy	Nigeria, Senegal, Gambia, Ghana, Mali, Côte d'Ivoire, Eritrea, Other	Nigeria, Senegal, Gambia, Ghana, Mali, Côte d'Ivoire, Eritrea, Other	67	NS	NS	4
RoufKhawaja 2020	uncontrolled, non-random, clinical study	India	Indian-held Kashmir	India	India	40	40	0	1
Saab 2003	cross-sectional	Lebanon	Lebanon	Lebanon	Lebanon	91	NS	NS	3
Saadi 2021	cross-sectional	United States	United States	Uganda	Uganda	1	1	0	1
Sachs 2008	cross-sectional	United States	India	China	China	83	NS	NS	13
Sanders 2009	cross-sectional	United States	United States	Albania, Eritrea, Iraq, Sri Lanka, Ecuador, Angola, Bulgaria, Somalia, Turkey, Peru, Cameroon, Serbia, Sudan, Congo DRC, Yugoslavia, Congo DRC, Ethiopia, Guinea, Kenya, Liberia, Sierra Leone, Togo, Uganda	Albania, Eritrea, Iraq, Sri Lanka, Ecuador, Angola, Bulgaria, Somalia, Turkey, Peru, Cameroon, Serbia, Sudan, Congo DRC, Yugoslavia, Congo DRC, Ethiopia, Guinea, Kenya, Liberia, Sierra Leone, Togo, Uganda	58	41	17	8
Sangmo 2020	cross-sectional	United States	United States	El Salvador, Honduras, Guatemala, Ecuador, Burkina Faso, Gambia, Côte d'Ivoire, Mali	El Salvador, Honduras, Guatemala, Ecuador, Burkina Faso, Gambia, Côte d'Ivoire, Mali	18	NS	NS	6
Savnik 2000	case-control	Denmark	Denmark	Middle East, South America	Middle East, South America	12	10	2	1
Savy 2008	case-report	Australia	Australia	Iraq	Iraq	1	1	0	2

© 2023 Milewski A et al. *JAMA Network Open.*

Study ID	Study design	Region(s) hosting researchers	Region(s) where researchers encountered population	Location of torture	Nationality of tortured individuals	Total tortured individuals (N)	Men (N)	Women (N)	Unique torture methods (N)
Schnyder 2015	uncontrolled, unblinded, non-randomized clinical study	Switzerland, Australia	Switzerland	Turkey, Iran, Sri Lanka, Bosnia and Herzegovina, Iraq, Afghanistan, Other	Turkey, Iran, Sri Lanka, Bosnia and Herzegovina, Iraq, Afghanistan, Other	103	NS	NS	1
Sen 2018	qualitative	Denmark	India	India	India	1	0	1	1
Singh 2012	qualitative	United States	Nepal	Nepal	Nepal	6	6	0	3
SmithFawzi 1997	cross-sectional	United States	United States	Vietnam	Vietnam	51	NS	NS	4
Somnier 1986	case-series	Denmark	Denmark	Latin America	Latin America	1	1	0	12
Song 2015	cross-sectional	United States, Netherlands	United States	Iraq, Iran, Eritrea, Other	Iraq, Iran, Eritrea, Other	278	126	152	8
Sutker 1990	cross-sectional/case-series	United States	United States	North Korea	United States	20	20	0	8
Tamblyn 2011	cross-sectional	United States	United States	Africa, Other	Africa, Other	58	41	17	3
Thomsen 2000	cross-sectional	Denmark	Denmark	Middle East	Middle East	18	18	0	8
Torp-Pedersen 2009	case-control	Denmark	Denmark	Middle East	Middle East	12	12	0	1
Tran 2020	cross-sectional	United States	United States	Asia, Africa, Latin America, Europe, Other	Asia, Africa, Latin America, Europe, Other	101	50	51	4
Tsai 2012	cross-sectional	United States	NA‡	Sudan	Sudan	292	NS	NS	14
Unuvar 2014	case-series	Turkey	Turkey	Turkey	Turkey	3	3	0	20
VanOmmeren 1998	cross-sectional	Nepal	Nepal	Bhutan	Bhutan	3212	NS	NS	5
VanOmmeren 2001	case-control	Nepal, United States	Nepal	Bhutan	Bhutan	418	324	94	20
VanVelsen 1996	cross-sectional	United Kingdom	United Kingdom	Middle East, Turkey, Africa, Latin America, Europe, Asia	Middle East, Turkey, Africa, Latin America, Europe, Asia	60	51	9	18

© 2023 Milewski A et al. *JAMA Network Open*.

Study ID	Study design	Region(s) hosting researchers	Region(s) where researchers encountered population	Location of torture	Nationality of tortured individuals	Total tortured individuals (N)	Men (N)	Women (N)	Unique torture methods (N)
VillerHansen 2017	cross-sectional, qualitative, uncontrolled unblinded unrandomized clinical study	Denmark, Tunisia, Iraq, Jordan, Lebanon, Sudan, Palestinian Territory, Syria	Tunisia, Iraq, Jordan, Lebanon, Sudan, Palestinian Territory, Syria	Syria, Iraq, Sudan, Tunisia, Libya, Jordan, Palestinian Territory, Egypt, Other	Syria, Iraq, Sudan, Tunisia, Libya, Jordan, Palestinian Territory, Egypt, Other	82	NS	NS	10
Vohra 2019	cross-sectional	India	India	India	India	19	NS	NS	5
Vrca 1996	cross-sectional	Croatia	Croatia	Yugoslavia	Yugoslavia	14	14	0	1
Wang 2009	cross-sectional	Denmark, Bangladesh	Bangladesh	Bangladesh	Bangladesh	171	NS	NS	12
Weinstein 1996	case-report	United States	United States	NS	NS	1	0	1	13
Weisaeth 1989	serial cross-sectional/case-series?/uncontrolled, unblinded, non-randomized clinical study	Norway	Norway	Libya	Norway	7	7	0	2
Weishut 2015	cross-sectional, qualitative	Israel, United States	Israel	Israel	Palestinian Territory	60	60	0	6
Wenzel 2000	cross-sectional, case report	Austria	Austria	Turkey, Iran, Iraq, Bosnia and Herzegovina, Bangladesh, Nigeria, Somalia, Albania	Turkey, Iran, Iraq, Bosnia and Herzegovina, Bangladesh, Nigeria, Somalia, Albania	72	70	2	5
Westermeyer 2011	cross-sectional	United States	United States	Ethiopia, Somalia	Ethiopia, Somalia	405	NS	NS	34
Wikholm 2020	cross-sectional	United States	United States	Guinea, Burkina Faso, Mali, Nigeria, Kenya, Sierra Leone, Côte d'Ivoire, Egypt, Gambia, Malawi, Chad, Djibouti, Senegal, Ethiopia, Sudan,	Guinea, Burkina Faso, Mali, Nigeria, Kenya, Sierra Leone, Côte d'Ivoire, Egypt, Gambia, Malawi, Chad, Djibouti, Senegal, Ethiopia,	119	0	119	2

© 2023 Milewski A et al. *JAMA Network Open.*

Study ID	Study design	Region(s) hosting researchers	Region(s) where researchers encountered population	Location of torture	Nationality of tortured individuals	Total tortured individuals (N)	Men (N)	Women (N)	Unique torture methods (N)
				Eritrea, Gabon, Liberia, Saudi Arabia, Somalia, Uganda, Zambia	Sudan, Eritrea, Gabon, Liberia, Saudi Arabia, Somalia, Uganda, Zambia				
Williams 2010	cross-sectional	United Kingdom	United Kingdom	Congo DRC, Turkey, Eritrea, Iran, Ethiopia, Iraq	Congo DRC, Turkey, Eritrea, Iran, Ethiopia, Iraq	178	115	63	9
Wilson 2013	cross-sectional (not case-control, because they looked at prevalence)	Ireland	Ireland	NS	NS	17	NS	NS	5
WOLF 1947	qualitative, testimonial, case-series?	United States	United States	Philippines, Japan	United States	2	2	0	2
Womersley 2018	case-report, case-series, qualitative	Switzerland	Greece	India	India	1	1	0	5
Young 2008	case-report	United Kingdom	United Kingdom	Middle East	Middle East	1	1	0	1
Zandieh 2016	case-control	Austria	Austria	Iran, Kurdistan, Turkey	Iran, Kurdistan, Turkey	9	7	2	5
Zech 2017	cross-sectional	United States	Peru	Peru	Peru	874	NS	NS	13

* NS = Not Specified
† Not Applicable; researchers used an online questionnaire
‡ Not Applicable; researchers reviewed records
§ Not Applicable; National Survey
Throughout the main text and the supplemental material, countries and territories are referred to by their modern names. If a country or territory that no longer exists was referenced in an article and could be ascribed entirely to a single modern country or territory, then the modern name was used. If a country or territory had instead been split into more than one modern country or territory, then single countries or territories were not ascribed and that data could not be used for mapping the geographic distributions of torture methods. If only part of a country or territory was referenced, then the entire modern country or territory was assigned. If regions were referenced that encompassed parts or all of multiple countries or territories, then that data could not be used for mapping the geographic distributions of the torture methods at the country level, but could be used for mapping at the level of United Nations subregions if a subregion could clearly be assigned. In cases of disputed territories, we followed the United Nations' classification. If torture was reported for a military base, then the country administering the base was assigned.

© 2023 Milewski A et al. *JAMA Network Open.*

eTable 3. Top Torture Methods as Ranked by a Consensus of 3 Strategies

**Always in Top 5** (at least 86 studies, 41 countries, and an average frequency of 0.188)<br>*Account for 31.3% of reported tortures, on average*
Beating or blunt-force trauma[a]
Other psychological torture[b]
Verbal abuse or threats
**Always in Top 10** (at least 71 studies, 28 countries, and an average frequency of 0.142)<br>*Account for 44.6% of reported tortures, on average*
Electrical torture
Witnessing torture
Isolation or solitary confinement
**Always in Top 15** (at least 60 studies, 23 countries, and an average frequency of 0.093)<br>*Account for 58.2% of reported tortures, on average*
Other physical torture
Starvation or dehydration
Sexual assault or abuse
Foot whipping
**Always in Top 20** (at least 49 studies, 18 countries, and an average frequency of 0.069)<br>*Account for 79.8% of reported tortures, on average*
Suspension
Burning
Asphyxiation or suffocation
Sharp objects or penetrating trauma
Rape
Blindfolding
Stripping naked
Binding or restricted movement
Other forced positions
**In Top 20 for 2 of 3 Strategies**<br>*Account for 84.0% of reported tortures, on average*
Prevention of hygiene or exposure to infested surroundings[c]
Sham executions[d]

[a]Ranked number 1 by all strategies.
[b]Ranked number 2 by all strategies.
[c]Ranked number 14 by average frequency,19 by number of countries, and 23 by number of studies.
[d]Ranked number 20 by number of studies and number of countries; ranked 21 by average frequency.
No method of torture ranked among the top 20 for only one ranking strategy.

© 2023 Milewski A et al. *JAMA Network Open.*

Comp. rank	Torture method	Number studies	Number countries	Avgerage frequency %
1	Beating or blunt-force trauma	208	59	62.4
2	Other psychological torture	132	48	31.6
3	Verbal abuse or threats	86	41	18.8
4	Electrical torture	114	28	17.2
5	Other physical torture	60	40	14.3
6	Witnessing torture	84	29	16.6
7	Isolation or solitary confinement	71	29	14.2
8	Sexual assault or abuse	76	26	9.3
9	Burning	84	25	6.9
10	Starvation or dehydration	65	26	12.7
11	Foot whipping	65	23	12.6
12	Suspension	75	21	11.0
13	Sharp objects or penetrating trauma	65	24	7.8
14	Rape	61	24	8.3
15	Asphyxiation or suffocation	68	21	8.9
16	Blindfolding	49	23	10.4
17	Stripped naked	51	23	8.0
18	Binding or restricted movement	51	21	8.3
19	Other forced positions	57	18	8.0
20	Prevention of hygiene	45	20	8.5
21	Sham executions	48	18	6.7
22	Water torture	47	15	5.8
23	Exposure to extreme hot or cold	39	17	5.7
24	Other sexual torture	44	15	6.4
25	Sleep deprivation	35	16	6.2
26	Forced prolonged standing	24	14	4.6
27	Deprivation of medical care	26	12	3.7
28	Gunshot	24	14	1.7
29	Denailing	27	12	1.2
30	Genital trauma	24	10	2.5
31	Pulling or dragging	21	9	3.0
32	Whipping	22	8	3.7
33	Exposure to bright light	16	10	2.1
34	FGM/C	8	12	1.3
35	Other sensory torture	19	8	2.1
36	Chemicals or irritants	16	9	1.7
37	Pharmacological torture	13	6	0.2
38	Loud noises	9	5	1.4
39	Removal of teeth	13	4	0.8
40	Muscle crushing with roller	9	4	1.0
41	Fluctuating interrogator attitude	6	4	0.8
42	Stretching extremities	7	3	0.9
43	Deprivation of light	5	3	0.5
44	Sexual enslavement	2	1	0.2
45	Stoning	1	0	0.1

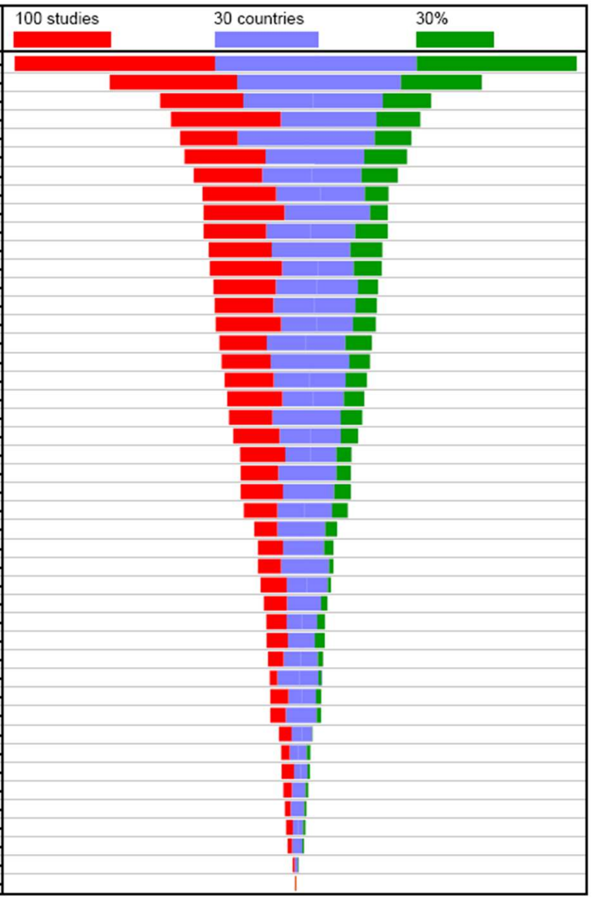

eFigure 3. Ranking of All Torture Methods

Three disparate strategies were used to rank the investigated torture methods: 1. The number of studies that reported the method. 2. The number of countries wherein the method was reported to have been perpetrated. 3. The average frequency of the method across all studies. A composite measure–represented in the graph by the total width of the bar, i.e. the sum of the red, blue, and green bars–was formed by adding the values for the three ranking strategies after normalizing each to the maximum value found for each strategy (the number of studies for a torture method was divided by 208, the number of countries by 59, and the average frequency by 62.4, and the three resulting dividends were summed). Sorting the torture methods from greatest to least according to their composite measures yields the composite (Comp.) rank shown. Strong positive associations were found between the average frequencies of the individual torture methods and the number of studies reporting the method (R=0.94), between the average frequencies and the number of countries wherein the methods were perpetrated (R=0.89), and between the number of studies reporting the methods and the number of countries wherein the methods were perpetrated (R=0.93).

© 2023 Milewski A et al. *JAMA Network Open.*

eTable 4. Top 10 Torture Methods Estimated by Pooled Frequencies

Torture	Frequency	S.E.M.
Beating or blunt-force trauma	0.917	0.000858822
Binding or restricted movement	0.547	0.00154663
Asphyxiation or suffocation	0.477	0.001551722
Electrical torture	0.269	0.001378307
Burning	0.09	0.000889992
Sharp objects or penetrating trauma	0.062	0.000747122
Foot whipping	0.033	0.000558428
Suspension	0.029	0.000520285
Starvation or dehydration	0.026	0.000496564
Other forced positions	0.021	0.000449582

© 2023 Milewski A et al. *JAMA Network Open.*

eTable 5. Countries in Which Individuals Reported That Torture Occurred

Country	N	Country	N	Country	N	Country	N
Afghanistan	14	Djibouti	2	Kuwait	1	Rwanda	2
Albania	4	Dominican Republic	1	Kyrgyzstan	1	Saudi Arabia	2
Algeria	3	Ecuador	4	Laos	1	Senegal	4
Angola	4	Egypt	7	Lebanon	10	Serbia#	8
Argentina	3	El Salvador	6	Liberia	4	Sierra Leone	10
Azerbaijan	1	Eritrea	9	Libya	5	Somalia	14
Bangladesh	6	Ethiopia	13	Madagascar	1	South Africa	7
Belarus	2	Gabon	2	Malawi	2	South Korea	1
Benin	1	Gambia	5	Malaysia	1	Spain	10
Bhutan	3	Georgia	1	Mali	6	Sri Lanka	16
Bolivia	2	Germany	2	Mauritania	3	Sudan	11
Bosnia and Herzegovina	12	Ghana	3	Mauritius	1	Suriname	1
Bulgaria	1	Greece	5	Mexico	5	Syria	12
Burkina Faso	5	Guatemala	5	Mongolia	1	Tanzania	2
Burundi	2	Guinea	10	Montenegro	2	Thailand	1
Cambodia	3	Guinea-Bissau	2	Morocco	4	Timor-Leste	1
Cameroon	10	Honduras	4	Myanmar	2	Togo	7
Central African Republic	2	India+	20	Nepal	3	Tunisia	2
Chad	4	Indonesia	3	Nigeria	9	Turkey	25
Chile	11	Iran	25	North Korea	1	Uganda	12
China	11	Iraq	30	Pakistan^	8	Ukraine	1
Colombia	3	Ireland	2	Palestinian Territory	6	United Kingdom	1
Congo	1	Israel	3	Peru	5	United States*	2
Congo DRC	14	Japan	3	Philippines	3	Uruguay	3
Côte d'Ivoire	8	Jordan	1	Romania	1	Vietnam	6
Croatia	7	Kazakhstan	1	Russian Federation	6	Zambia	1
Cuba	1	Kenya	4				

N = number of studies. For 59 studies, individual countries could not be identified because the location of torture was either not specified or a larger region rather than individual countries was specified. In one study, individuals reported being tortured in Western Sahara, which the UN considers to be a non-self-governing territory.
*Includes 1 study wherein the individuals reported being tortured in Guantanamo Bay Naval Base
#Includes 6 studies wherein the individuals reported being tortured in Kosovo, which the UN considers part of Serbia.
+Includes 4 studies wherein the individuals reported being tortured in India-administered Kashmir.
^Includes 1 study wherein the individuals reported being tortured in Pakistan-administered Kashmir.
~Includes 9 studies wherein the individuals reported being tortured in Tibet, which the UN considers part of China.

© 2023 Milewski A et al. *JAMA Network Open.*

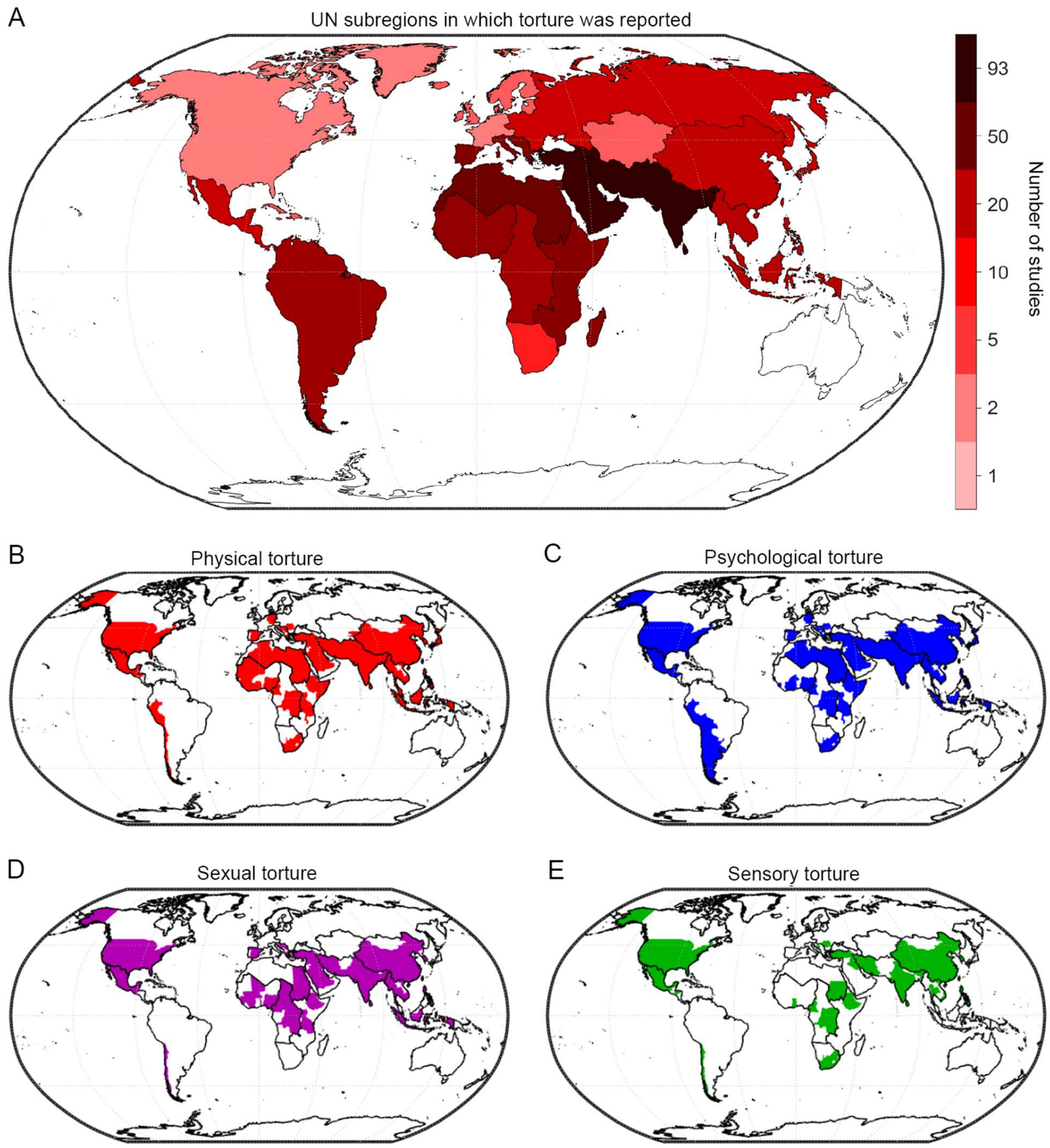

eFigure 4. UN Subregions in Which Torture Occurred and Minimal Geographic Extent of Torture Categories

Individuals reported that they were subjected to torture in 18 UN subregions. Darker shades of red indicate that a greater number of studies reported torture in that UN subregion. Torture was not reported for every country within any UN subregion, see **Figure 1a** and **Table S3** for the individual countries wherein torture was clearly reported to have occurred. (B-E) The shaded areas indicate the minimal geographic extent for any form of physical torture (B), psychological torture (C), sexual torture (D), or sensory torture (E). United Nations subregions are outlined and individual countries are shaded if the torture category was reported to have occurred there.

© 2023 Milewski A et al. *JAMA Network Open.*

eTable 6. UN Subregions in Which Individuals Reported That Torture Occurred

UN Sub Region	N	UN Sub Region	N	UN Sub Region	N
Caribbean	3	Eastern Europe	17	Southern Africa	7
Central Africa	27	Northern America	2	Southern Asia	93
Central America	18	Northern Africa	47	Southern Europe	38
Central Asia	3	Northern Europe	3	Western Africa	32
Eastern Africa	35	South America	28	Western Asia	85
Eastern Asia	19	Southeastern Asia	21	Western Europe	2

N = number of studies
No studies reported torture in the other four UN sub-regions: Australia and New Zealand, Melanesia, Micronesia, and Polynesia.

© 2023 Milewski A et al. *JAMA Network Open.*

eTable 7. Countries Where Torture Mechanisms Were Reported to Occur, Organized by Country

Country	No. of studies*	Torture methods (number of studies reporting each torture method)
Afghanistan	14	Beating or blunt-force trauma (1), Sharp objects or penetrating trauma (1), Other physical torture (1), Verbal abuse or threats (1), Isolation or solitary confinement (1)
Albania	4	Other psychological torture (1)
Algeria	3	Beating or blunt-force trauma (1), Electrical torture (1), Water torture (1), Asphyxiation or suffocation (1), Binding or restricted movement (1), Other physical torture (1), Removal of teeth (1), Verbal abuse or threats (1), Witnessing torture (1)
Angola	4	Unclear
Argentina	3	Other psychological torture (1)
Azerbaijan	1	Unclear
Bangladesh	6	Beating or blunt-force trauma (2), Electrical torture (1), Burning (1), Water torture (1), Asphyxiation or suffocation (1), Foot whipping (1), Other forced positions (1), Denailing (1), Sexual assault or abuse (1), Verbal abuse or threats (1), Other psychological torture (2), Blindfolding (1)
Belarus	2	Unclear
Benin	1	Unclear
Bhutan	3	Beating or blunt-force trauma (2), Pulling or dragging (1), Asphyxiation or suffocation (1), Suspension (1), Foot whipping (1), Starvation or dehydration (1), Deprivation of medical care (1), Other forced positions (1), Binding or restricted movement (1), Forced prolonged standing (1), Denailing (1), Chemicals or irritants (1), Exposure to extreme hot or cold (1), Other sensory torture (1), Stripped naked (1), Verbal abuse or threats (1), Other psychological torture (2), Isolation or solitary confinement (2), Prevention of hygiene or exposure to infested surroundings (2), Sleep deprivation (2)
Bolivia	2	Other psychological torture (1)
Bosnia and Herzegovina	12	Beating or blunt-force trauma (1), Other forced positions (1), Forced prolonged standing (1), Other physical torture (1), Exposure to extreme hot or cold (1), Rape (2), Verbal abuse or threats (1), Witnessing torture (1), Other psychological torture (3), Isolation or solitary confinement (1)
Bulgaria	1	Unclear
Burkina Faso	5	FGM/C (1), Verbal abuse or threats (1)
Burundi	2	Beating or blunt-force trauma (1)
Cambodia	3	Beating or blunt-force trauma (2), Burning (2), Asphyxiation or suffocation (1), Sharp objects or penetrating trauma (2), Starvation or dehydration (1), Binding or restricted movement (1), Other physical torture (1), Rape (1), Other psychological torture (1), Blindfolding

© 2023 Milewski A et al. *JAMA Network Open*.

Country	No. of studies*	Torture methods (number of studies reporting each torture method)
		(1)
Cameroon	10	Beating or blunt-force trauma (2), Electrical torture (1), Pulling or dragging (1), Foot whipping (1), Other physical torture (1), Exposure to bright light (1), FGM/C (1), Other sexual torture (1), Verbal abuse or threats (1), Witnessing torture (1)
Central African Republic	2	FGM/C (1)
Chad	4	FGM/C (1)
Chile	11	Beating or blunt-force trauma (4), Electrical torture (5), Burning (3), Water torture (2), Sharp objects or penetrating trauma (2), Suspension (2), Foot whipping (1), Whipping (1), Starvation or dehydration (1), Deprivation of medical care (1), Other forced positions (1), Binding or restricted movement (1), Other physical torture (1), Denailing (1), Pharmacological torture (1), Chemicals or irritants (1), Exposure to extreme hot or cold (1), Exposure to bright light (1), Sexual assault or abuse (2), Sham executions (1), Stripped naked (2), Verbal abuse or threats (2), Witnessing torture (1), Other psychological torture (4), Isolation or solitary confinement (1), Blindfolding (2), Prevention of hygiene or exposure to infested surroundings (1), Sleep deprivation (2)
China	11	Beating or blunt-force trauma (4), Electrical torture (3), Burning (3), Water torture (2), Asphyxiation or suffocation (1), Sharp objects or penetrating trauma (3), Gunshot (1), Suspension (3), Foot whipping (1), Starvation or dehydration (3), Deprivation of medical care (1), Other forced positions (2), Binding or restricted movement (3), Forced prolonged standing (1), Other physical torture (1), Denailing (1), Exposure to extreme hot or cold (3), Exposure to bright light (1), Rape (2), Sexual assault or abuse (3), Other sexual torture (1), Sham executions (1), Stripped naked (4), Verbal abuse or threats (3), Witnessing torture (1), Other psychological torture (2), Isolation or solitary confinement (3), Prevention of hygiene or exposure to infested surroundings (1), Sleep deprivation (2)
Colombia	3	Unclear
Congo	1	Unclear
Congo DRC	13	Beating or blunt-force trauma (2), Electrical torture (1), Burning (1), Sharp objects or penetrating trauma (1), Starvation or dehydration (1), Other physical torture (1), Exposure to bright light (1), Rape (1), Sexual assault or abuse (1), FGM/C (1), Sham executions (1), Verbal abuse or threats (1), Witnessing torture (1), Prevention of hygiene or exposure to infested surroundings (1)
Côte d'Ivoire	8	Beating or blunt-force trauma (1), Other physical torture (1), Chemicals or irritants (1), FGM/C (1), Other psychological torture (1)
Croatia	7	Beating or blunt-force trauma (1), Other physical torture (1), Rape (2), Verbal abuse or threats (1), Other psychological torture (1), Isolation or solitary confinement (1)
Cuba	1	Unclear
Cuba-	1	Beating or blunt-force trauma (1), Starvation or dehydration (1), Other forced positions (1), Exposure to extreme hot or cold (1),

© 2023 Milewski A et al. *JAMA Network Open.*

Country	No. of studies*	Torture methods (number of studies reporting each torture method)
Guantánamo Bay		Sexual assault or abuse (1), Sham executions (1), Stripped naked (1), Verbal abuse or threats (1), Other psychological torture (1), Isolation or solitary confinement (1), Blindfolding (1), Sleep deprivation (1)
Djibouti	2	Unclear
Dominican Republic	1	Unclear
Ecuador	4	Unclear
Egypt	7	Beating or blunt-force trauma (1), Electrical torture (1), Burning (1), Pulling or dragging (1), Sharp objects or penetrating trauma (1), Gunshot (1), Other sexual torture (1), Verbal abuse or threats (1)
El Salvador	6	Witnessing torture (1), Other psychological torture (1), Blindfolding (1)
Eritrea	9	Verbal abuse or threats (1)
Ethiopia	13	Beating or blunt-force trauma (1), Electrical torture (1), Burning (1), Water torture (1), Pulling or dragging (1), Asphyxiation or suffocation (1), Sharp objects or penetrating trauma (1), Suspension (1), Foot whipping (1), Whipping (1), Starvation or dehydration (1), Deprivation of medical care (1), Other forced positions (1), Binding or restricted movement (1), Stretching extremities (1), Denailing (1), Removal of teeth (1), Pharmacological torture (1), Exposure to bright light (1), Loud noises (1), Deprivation of light (1), Other sensory torture (1), Rape (1), Sexual assault or abuse (1), Genital trauma (1), Other sexual torture (1), Sham executions (1), Stripped naked (1), Verbal abuse or threats (1), Fluctuating interrogator attitude (1), Witnessing torture (1), Other psychological torture (1), Isolation or solitary confinement (1), Blindfolding (1), Prevention of hygiene or exposure to infested surroundings (1), Sleep deprivation (1)
Gabon	2	Unclear
Gambia	5	Other physical torture (1)
Georgia	1	Unclear
Germany	2	Other physical torture (1), Verbal abuse or threats (1), Witnessing torture (1), Other psychological torture (2), Isolation or solitary confinement (1)
Ghana	3	Beating or blunt-force trauma (1), Burning (1), Sharp objects or penetrating trauma (1), Whipping (1), Exposure to bright light (1), Other psychological torture (1), Isolation or solitary confinement (1)
Greece	5	Beating or blunt-force trauma (2), Electrical torture (3), Suspension (2), Foot whipping (2), Starvation or dehydration (1), Other physical torture (2), Sexual assault or abuse (1), Genital trauma (2), Sham executions (2), Other psychological torture (2), Isolation or solitary confinement (2), Sleep deprivation (2)

© 2023 Milewski A et al. *JAMA Network Open*.

Country	No. of studies*	Torture methods (number of studies reporting each torture method)
Guatemala	5	Beating or blunt-force trauma (1), Burning (1), Rape (1), Witnessing torture (1), Other psychological torture (1)
Guinea	10	Beating or blunt-force trauma (1), Other physical torture (1), FGM/C (1)
Guinea-Bissau	2	Unclear
Honduras	4	Beating or blunt-force trauma (1), Starvation or dehydration (1), Exposure to extreme hot or cold (1), Stripped naked (1), Verbal abuse or threats (1), Other psychological torture (1), Isolation or solitary confinement (1), Blindfolding (1), Prevention of hygiene or exposure to infested surroundings (1)
India	20	Beating or blunt-force trauma (11), Electrical torture (7), Burning (2), Muscle crushing with roller (3), Sharp objects or penetrating trauma (2), Suspension (4), Foot whipping (1), Other forced positions (4), Stretching extremities (3), Other physical torture (4), Chemicals or irritants (1), Exposure to extreme hot or cold (1), Other sexual torture (4), Stripped naked (1), Verbal abuse or threats (2), Other psychological torture (3), Blindfolding (2), Prevention of hygiene or exposure to infested surroundings (1)
Indonesia	3	Beating or blunt-force trauma (1), Electrical torture (1), Starvation or dehydration (1), Binding or restricted movement (1), Rape (1), Other sexual torture (1), Stripped naked (1), Other psychological torture (1)
Iran	25	Beating or blunt-force trauma (4), Electrical torture (1), Burning (1), Suspension (1), Foot whipping (3), Whipping (1), Other physical torture (4), Removal of teeth (1), Deprivation of light (1), Sexual assault or abuse (1), Sham executions (1), Verbal abuse or threats (2), Witnessing torture (1), Other psychological torture (3), Isolation or solitary confinement (2), Prevention of hygiene or exposure to infested surroundings (1)
Iraq	30	Beating or blunt-force trauma (5), Electrical torture (5), Burning (2), Water torture (2), Asphyxiation or suffocation (1), Suspension (3), Foot whipping (5), Whipping (2), Starvation or dehydration (5), Deprivation of medical care (3), Binding or restricted movement (1), Forced prolonged standing (2), Other physical torture (1), Denailing (1), Pharmacological torture (1), Exposure to extreme hot or cold (2), Loud noises (2), Rape (3), Sexual assault or abuse (2), Genital trauma (2), Sham executions (1), Stripped naked (3), Verbal abuse or threats (5), Witnessing torture (3), Other psychological torture (8), Isolation or solitary confinement (3), Blindfolding (2), Prevention of hygiene or exposure to infested surroundings (4), Sleep deprivation (3)
Ireland	2	Unclear
Israel	3	Beating or blunt-force trauma (3), Electrical torture (2), Other physical torture (1), Exposure to extreme hot or cold (1), Exposure to bright light (1), Loud noises (1), Rape (1), Sexual assault or abuse (1), Genital trauma (1), Other sexual torture (1), Stripped naked (2), Verbal abuse or threats (2), Witnessing torture (1), Isolation or solitary confinement (1), Blindfolding (1), Sleep deprivation (1)
Japan	3	Beating or blunt-force trauma (2), Starvation or dehydration (1), Other psychological torture (1)
Jordan	1	Unclear

© 2023 Milewski A et al. *JAMA Network Open*.

Country	No. of studies*	Torture methods (number of studies reporting each torture method)
Kazakhstan	1	Unclear
Kenya	4	Unclear
Kuwait	1	Electrical torture (1)
Kyrgyzstan	1	Beating or blunt-force trauma (1), Electrical torture (1), Asphyxiation or suffocation (1), Sharp objects or penetrating trauma (1), Gunshot (1), Foot whipping (1), Other psychological torture (1)
Laos	1	Unclear
Lebanon	9	Beating or blunt-force trauma (3), Electrical torture (2), Burning (1), Water torture (1), Asphyxiation or suffocation (2), Sharp objects or penetrating trauma (1), Suspension (1), Foot whipping (1), Starvation or dehydration (1), Other forced positions (1), Other physical torture (2), Sexual assault or abuse (2), Stripped naked (1), Verbal abuse or threats (1), Witnessing torture (1), Other psychological torture (3), Isolation or solitary confinement (2), Blindfolding (1)
Liberia	4	Other physical torture (1)
Libya	5	Beating or blunt-force trauma (2), Verbal abuse or threats (1), Witnessing torture (1)
Madagascar	1	Unclear
Malawi	2	Beating or blunt-force trauma (1), Verbal abuse or threats (1)
Malaysia	1	Beating or blunt-force trauma (1), Foot whipping (1)
Mali	6	Other physical torture (1), FGM/C (1)
Mauritania	3	Other physical torture (1)
Mauritius	1	Unclear
Mexico	5	Beating or blunt-force trauma (3), Electrical torture (3), Burning (2), Water torture (1), Asphyxiation or suffocation (3), Sharp objects or penetrating trauma (2), Gunshot (1), Starvation or dehydration (2), Other forced positions (1), Binding or restricted movement (1), Other sensory torture (1), Rape (1), Genital trauma (1), Other sexual torture (1), Stripped naked (2), Verbal abuse or threats (2), Witnessing torture (1), Other psychological torture (2), Isolation or solitary confinement (1), Blindfolding (2)
Mongolia	1	Unclear
Montenegro	2	Unclear
Morocco	4	Unclear
Multi	72	Unclear

© 2023 Milewski A et al. *JAMA Network Open.*

Country	No. of studies*	Torture methods (number of studies reporting each torture method)
Myanmar	2	Beating or blunt-force trauma (2), Electrical torture (1), Burning (2), Water torture (1), Asphyxiation or suffocation (1), Muscle crushing with roller (1), Sharp objects or penetrating trauma (1), Gunshot (1), Suspension (1), Chemicals or irritants (1), Sexual assault or abuse (1), Sham executions (1)
Nepal	3	Beating or blunt-force trauma (2), Electrical torture (1), Water torture (1), Asphyxiation or suffocation (1), Muscle crushing with roller (1), Sharp objects or penetrating trauma (1), Suspension (1), Foot whipping (1), Starvation or dehydration (1), Deprivation of medical care (1), Other physical torture (1), Denailing (1), Removal of teeth (1), Rape (1), Sexual assault or abuse (1), Verbal abuse or threats (1), Other psychological torture (1), Isolation or solitary confinement (1), Prevention of hygiene or exposure to infested surroundings (1)
Nigeria	9	Beating or blunt-force trauma (1), Sharp objects or penetrating trauma (1), Binding or restricted movement (1), Other physical torture (2), Rape (1), Sexual assault or abuse (1), FGM/C (1), Stripped naked (1), Verbal abuse or threats (1), Other psychological torture (2), Blindfolding (1)
North Korea	1	Beating or blunt-force trauma (1), Other forced positions (1), Other physical torture (1), Verbal abuse or threats (1), Witnessing torture (1), Other psychological torture (1), Isolation or solitary confinement (1), Prevention of hygiene or exposure to infested surroundings (1)
Not Specified	36	Unclear
Pakistan	8	Beating or blunt-force trauma (3), Burning (2), Muscle crushing with roller (1), Sharp objects or penetrating trauma (2), Foot whipping (1), Forced prolonged standing (1), Other physical torture (1), Chemicals or irritants (1), Other sexual torture (1), Other psychological torture (1)
Palestinian Territory	6	Beating or blunt-force trauma (2), Electrical torture (1), Burning (1), Suspension (1), Whipping (1), Starvation or dehydration (1), Forced prolonged standing (1), Exposure to extreme hot or cold (1), Rape (1), Sexual assault or abuse (1), Genital trauma (1), Verbal abuse or threats (1), Other psychological torture (1), Isolation or solitary confinement (1)
Peru	5	Beating or blunt-force trauma (3), Electrical torture (1), Burning (1), Asphyxiation or suffocation (1), Sharp objects or penetrating trauma (1), Suspension (1), Starvation or dehydration (1), Other physical torture (1), Pharmacological torture (1), Sham executions (1), Stripped naked (1), Verbal abuse or threats (1), Other psychological torture (2), Prevention of hygiene or exposure to infested surroundings (1), Sleep deprivation (1)
Philippines	3	Beating or blunt-force trauma (2), Burning (1), Other forced positions (1), Binding or restricted movement (1), Chemicals or irritants (1), Exposure to extreme hot or cold (1), Rape (1), Sexual assault or abuse (1), Other sexual torture (1), Stripped naked (1), Verbal abuse or threats (1), Witnessing torture (1), Other psychological torture (1), Blindfolding (1), Prevention of hygiene or exposure to infested surroundings (1), Sleep deprivation (1)

© 2023 Milewski A et al. *JAMA Network Open.*

Country	No. of studies*	Torture methods (number of studies reporting each torture method)
Republic of Caucasus	1	Unclear
Romania	1	Beating or blunt-force trauma (1), Starvation or dehydration (1), Deprivation of medical care (1), Forced prolonged standing (1), Other physical torture (1), Exposure to extreme hot or cold (1), Deprivation of light (1), Sham executions (1), Verbal abuse or threats (1), Witnessing torture (1), Other psychological torture (1), Isolation or solitary confinement (1), Blindfolding (1), Sleep deprivation (1)
Russian Federation	6	Unclear
Rwanda	2	Beating or blunt-force trauma (1)
Saudi Arabia	2	Beating or blunt-force trauma (1), Asphyxiation or suffocation (1), Suspension (1), Foot whipping (1), Forced prolonged standing (1), Rape (1), Sexual assault or abuse (1), Sham executions (1), Other psychological torture (1), Isolation or solitary confinement (1), Sleep deprivation (1)
Senegal	4	Other physical torture (1)
Serbia	7	Beating or blunt-force trauma (1), Gunshot (1), Other physical torture (1), Sexual assault or abuse (1), Verbal abuse or threats (1), Witnessing torture (1), Other psychological torture (1)
Sierra Leone	10	Beating or blunt-force trauma (1), Burning (1), Foot whipping (1), Other physical torture (1), Rape (1), Sexual assault or abuse (1), Verbal abuse or threats (1), Witnessing torture (1)
Somalia	14	Beating or blunt-force trauma (1), Starvation or dehydration (1), Forced prolonged standing (1), Other physical torture (1), Sham executions (1), Other psychological torture (1)
South Africa	7	Beating or blunt-force trauma (3), Electrical torture (2), Asphyxiation or suffocation (1), Foot whipping (1), Starvation or dehydration (1), Other physical torture (1), Exposure to bright light (1), Isolation or solitary confinement (1)
South Korea	1	Beating or blunt-force trauma (1), Electrical torture (1), Water torture (1), Suspension (1), Deprivation of medical care (1), Binding or restricted movement (1), Denailing (1), Pharmacological torture (1), Exposure to extreme hot or cold (1), Other sensory torture (1), Other sexual torture (1), Sham executions (1), Stripped naked (1), Verbal abuse or threats (1), Fluctuating interrogator attitude (1), Witnessing torture (1), Other psychological torture (1), Isolation or solitary confinement (1), Blindfolding (1), Sleep deprivation (1)
Spain	10	Beating or blunt-force trauma (7), Electrical torture (5), Water torture (2), Pulling or dragging (2), Asphyxiation or suffocation (5), Sharp objects or penetrating trauma (1), Gunshot (1), Suspension (1), Foot whipping (1), Starvation or dehydration (2), Deprivation of medical care (2), Other forced positions (6), Binding or restricted movement (1), Forced prolonged standing (2), Other physical torture (3), Rape (1), Sexual assault or abuse (1), Genital trauma (1), Other sexual torture (2), Sham executions (3), Stripped naked (4), Verbal abuse or threats (1), Fluctuating interrogator attitude (1), Witnessing torture (2), Other psychological torture (5), Isolation or solitary

© 2023 Milewski A et al. *JAMA Network Open.*

Country	No. of studies*	Torture methods (number of studies reporting each torture method)
		confinement (3), Blindfolding (2), Prevention of hygiene or exposure to infested surroundings (1), Sleep deprivation (3)
Sri Lanka	16	Beating or blunt-force trauma (10), Electrical torture (6), Burning (7), Water torture (3), Pulling or dragging (3), Asphyxiation or suffocation (3), Sharp objects or penetrating trauma (5), Gunshot (2), Suspension (5), Foot whipping (2), Whipping (2), Starvation or dehydration (2), Deprivation of medical care (1), Other forced positions (1), Binding or restricted movement (5), Other physical torture (2), Denailing (4), Chemicals or irritants (3), Exposure to extreme hot or cold (1), Other sensory torture (1), Rape (5), Sexual assault or abuse (5), Genital trauma (2), FGM/C (1), Other sexual torture (2), Sham executions (1), Stripped naked (3), Verbal abuse or threats (4), Witnessing torture (2), Other psychological torture (9), Isolation or solitary confinement (2), Blindfolding (4), Prevention of hygiene or exposure to infested surroundings (2)
Sudan	11	Beating or blunt-force trauma (3), Burning (1), Gunshot (1), Suspension (1), Binding or restricted movement (1), Other physical torture (1), Exposure to bright light (1), Other sensory torture (1), Rape (1), Sexual assault or abuse (1), Genital trauma (1), FGM/C (1), Stripped naked (1), Verbal abuse or threats (1), Other psychological torture (1), Prevention of hygiene or exposure to infested surroundings (1)
Suriname	1	Unclear
Syria	12	Beating or blunt-force trauma (4), Foot whipping (1), Other forced positions (1), Binding or restricted movement (1), Forced prolonged standing (1), Rape (1), Stripped naked (1), Witnessing torture (2), Other psychological torture (2), Blindfolding (1)
Tanzania	2	Beating or blunt-force trauma (1), Pulling or dragging (1), Sharp objects or penetrating trauma (1), Gunshot (1), Suspension (1), Starvation or dehydration (1), Deprivation of medical care (1), Binding or restricted movement (1), Denailing (1), Rape (1), Sexual assault or abuse (1), Stripped naked (1), Verbal abuse or threats (1), Witnessing torture (1), Other psychological torture (1), Blindfolding (1), Prevention of hygiene or exposure to infested surroundings (1)
Thailand	1	Beating or blunt-force trauma (1), Burning (1), Asphyxiation or suffocation (1), Sharp objects or penetrating trauma (1), Gunshot (1), Other forced positions (1), Chemicals or irritants (1), Exposure to extreme hot or cold (1), Loud noises (1), Other sensory torture (1), Sexual assault or abuse (1), Stripped naked (1), Witnessing torture (1), Other psychological torture (1), Isolation or solitary confinement (1), Blindfolding (1), Prevention of hygiene or exposure to infested surroundings (1)
Timor-Leste	1	Beating or blunt-force trauma (1), Asphyxiation or suffocation (1), Sharp objects or penetrating trauma (1), Binding or restricted movement (1)
Togo	7	Other physical torture (2), FGM/C (1)
Tunisia	2	Unclear
Turkey	25	Beating or blunt-force trauma (9), Electrical torture (5), Burning (4), Water torture (3), Pulling or dragging (1), Asphyxiation or suffocation (5), Sharp objects or penetrating trauma (1), Gunshot (1), Suspension (4), Foot whipping (5), Starvation or dehydration (3),

© 2023 Milewski A et al. *JAMA Network Open.*

Country	No. of studies*	Torture methods (number of studies reporting each torture method)
		Deprivation of medical care (2), Other forced positions (1), Binding or restricted movement (6), Forced prolonged standing (3), Stretching extremities (1), Other physical torture (3), Denailing (2), Exposure to extreme hot or cold (2), Exposure to bright light (2), Loud noises (2), Other sensory torture (2), Rape (3), Sexual assault or abuse (4), FGM/C (1), Other sexual torture (1), Sham executions (4), Stripped naked (2), Verbal abuse or threats (2), Fluctuating interrogator attitude (1), Witnessing torture (3), Other psychological torture (7), Isolation or solitary confinement (4), Blindfolding (5), Prevention of hygiene or exposure to infested surroundings (4), Sleep deprivation (3)
Uganda	12	Beating or blunt-force trauma (5), Burning (1), Pulling or dragging (1), Sharp objects or penetrating trauma (2), Gunshot (2), Whipping (1), Starvation or dehydration (1), Other forced positions (1), Binding or restricted movement (2), Forced prolonged standing (1), Other physical torture (1), Rape (3), Sexual assault or abuse (1), Sexual enslavement (1), Other sexual torture (1), Verbal abuse or threats (1)
Ukraine	1	Unclear
United Kingdom	1	Unclear
United States	1	Unclear
Uruguay	3	Other psychological torture (1)
Vietnam	6	Beating or blunt-force trauma (3), Electrical torture (1), Water torture (2), Asphyxiation or suffocation (2), Gunshot (1), Suspension (1), Foot whipping (1), Starvation or dehydration (1), Binding or restricted movement (1), Forced prolonged standing (1), Other physical torture (1), Denailing (1), Pharmacological torture (1), Exposure to extreme hot or cold (1), Genital trauma (1), Sham executions (2), Witnessing torture (2), Other psychological torture (1), Isolation or solitary confinement (2), Blindfolding (1), Prevention of hygiene or exposure to infested surroundings (1), Sleep deprivation (1)
Western Sahara	1	Unclear
Zambia	1	Unclear

*Number of studies gives the number of papers that report torture occurred in the country. This number may not equal the sum of the number of papers for which specific torture methods could be determined: A single paper often studied torture victims from multiple countries and it was not possible to determine which victims were subjected to which torture methods.

© 2023 Milewski A et al. *JAMA Network Open.*

eTable 8. Countries Wherein Each Torture Method Was Reported to Occur, Organized by Torture Method

Torture method	No. of studies	No. of countries	No. of UN subregions	No. of individuals	Average frequency	Countries where torture was reported to occur (number of studies for each country)
Beating or blunt-force trauma	208	59	15	94967	0.624	Afghanistan (1), Algeria (1), Bangladesh (2), Bhutan (2), Bosnia and Herzegovina (1), Burundi (1), Cambodia (2), Cameroon (2), Chile (4), China (4), Congo DRC (2), Croatia (1), Côte d'Ivoire (1), Egypt (1), Ethiopia (1), Ghana (1), Greece (2), Guatemala (1), Guinea (1), Honduras (1), India (11), Indonesia (1), Iran (4), Iraq (5), Israel (3), Japan (2), Kyrgyzstan (1), Lebanon (3), Libya (2), Malawi (1), Malaysia (1), Mexico (3), Myanmar (2), Nepal (2), Nigeria (1), North Korea (1), Pakistan (3), Palestinian Territory (2), Peru (3), Philippines (2), Romania (1), Rwanda (1), Saudi Arabia (1), Serbia (1), Sierra Leone (1), Somalia (1), South Africa (3), South Korea (1), Spain (7), Sri Lanka (10), Sudan (3), Syria (4), Tanzania (1), Thailand (1), Timor-Leste (1), Turkey (9), Uganda (5), United States/Guantánamo Bay (1), Vietnam (3)
Electrical torture	114	28	12	27910	0.172	Algeria (1), Bangladesh (1), Cameroon (1), Chile (5), China (3), Congo DRC (1), Egypt (1), Ethiopia (1), Greece (3), India (7), Indonesia (1), Iran (1), Iraq (5), Israel (2), Kuwait (1), Kyrgyzstan (1), Lebanon (2), Mexico (3), Myanmar (1), Nepal (1), Palestinian Territory (1), Peru (1), South Africa (2), South Korea (1), Spain (5), Sri Lanka (6), Turkey (5), Vietnam (1)
Burning	84	25	11	9345	0.069	Bangladesh (1), Cambodia (2), Chile (3), China (3), Congo DRC (1), Egypt (1), Ethiopia (1), Ghana (1), Guatemala (1), India (2), Iran (1), Iraq (2), Lebanon (1), Mexico (2), Myanmar (2), Pakistan (2), Palestinian Territory (1), Peru (1), Philippines (1), Sierra Leone (1), Sri Lanka (7), Sudan (1), Thailand (1), Turkey (4), Uganda (1)
Water torture	47	15	9	752	0.058	Algeria (1), Bangladesh (1), Chile (2), China (2), Ethiopia (1), Iraq (2), Lebanon (1), Mexico (1), Myanmar (1), Nepal (1), South Korea (1), Spain (2), Sri Lanka (3), Turkey (3), Vietnam (2)
Pulling or dragging	21	9	6	1220	0.03	Bhutan (1), Cameroon (1), Egypt (1), Ethiopia (1), Spain (2), Sri Lanka (3), Tanzania (1), Turkey (1), Uganda (1)
Asphyxiation or suffocation	68	21	11	49399	0.089	Algeria (1), Bangladesh (1), Bhutan (1), Cambodia (1), China (1), Ethiopia (1), Iraq (1), Kyrgyzstan (1), Lebanon (2), Mexico (3), Myanmar (1), Nepal (1), Peru (1), Saudi Arabia (1), South Africa (1), Spain (5), Sri Lanka (3), Thailand (1), Timor-Leste (1), Turkey (5), Vietnam (2)
Muscle crushing with roller	9	4	2	189	0.01	India (3), Myanmar (1), Nepal (1), Pakistan (1)
Sharp objects or penetrating trauma	65	24	12	6385	0.078	Afghanistan (1), Cambodia (2), Chile (2), China (3), Congo DRC (1), Egypt (1), Ethiopia (1), Ghana (1), India (2), Kyrgyzstan (1), Lebanon (1), Mexico (2), Myanmar

© 2023 Milewski A et al. *JAMA Network Open.*

Torture method	No. of studies	No. of countries	No. of UN subregions	No. of individuals	Average frequency	Countries where torture was reported to occur (number of studies for each country)
						(1), Nepal (1), Nigeria (1), Pakistan (2), Peru (1), Spain (1), Sri Lanka (5), Tanzania (1), Thailand (1), Timor-Leste (1), Turkey (1), Uganda (2)
Gunshot	24	14	9	360	0.017	China (1), Egypt (1), Kyrgyzstan (1), Mexico (1), Myanmar (1), Serbia (1), Spain (1), Sri Lanka (2), Sudan (1), Tanzania (1), Thailand (1), Turkey (1), Uganda (2), Vietnam (1)
Suspension	75	21	8	2992	0.110	Bhutan (1), Chile (2), China (3), Ethiopia (1), Greece (2), India (4), Iran (1), Iraq (3), Lebanon (1), Myanmar (1), Nepal (1), Palestinian Territory (1), Peru (1), Saudi Arabia (1), South Korea (1), Spain (1), Sri Lanka (5), Sudan (1), Tanzania (1), Turkey (4), Vietnam (1)
Foot whipping	65	23	12	3463	0.126	Bangladesh (1), Bhutan (1), Cameroon (1), Chile (1), China (1), Ethiopia (1), Greece (2), India (1), Iran (3), Iraq (5), Kyrgyzstan (1), Lebanon (1), Malaysia (1), Nepal (1), Pakistan (1), Saudi Arabia (1), Sierra Leone (1), South Africa (1), Spain (1), Sri Lanka (2), Syria (1), Turkey (5), Vietnam (1)
Whipping	22	8	5	634	0.037	Chile (1), Ethiopia (1), Ghana (1), Iran (1), Iraq (2), Palestinian Territory (1), Sri Lanka (2), Uganda (1)
Starvation or dehydration	65	26	12	2718	0.127	Bhutan (1), Cambodia (1), Chile (1), China (3), Congo DRC (1), Ethiopia (1), Greece (1), Honduras (1), Indonesia (1), Iraq (5), Japan (1), Lebanon (1), Mexico (2), Nepal (1), Palestinian Territory (1), Peru (1), Romania (1), Somalia (1), South Africa (1), Spain (2), Sri Lanka (2), Tanzania (1), Turkey (3), Uganda (1), United States/Guantánamo Bay (1), Vietnam (1)
Deprivation of medical care	26	12	7	1556	0.037	Bhutan (1), Chile (1), China (1), Ethiopia (1), Iraq (3), Nepal (1), Romania (1), South Korea (1), Spain (2), Sri Lanka (1), Tanzania (1), Turkey (2)
Other forced positions	57	18	9	2217	0.080	Bangladesh (1), Bhutan (1), Bosnia and Herzegovina (1), Chile (1), China (2), Ethiopia (1), India (4), Lebanon (1), Mexico (1), North Korea (1), Philippines (1), Spain (6), Sri Lanka (1), Syria (1), Thailand (1), Turkey (1), Uganda (1), United States/Guantánamo Bay (1)
Binding or restricted movement	51	21	10	56631	0.083	Algeria (1), Bhutan (1), Cambodia (1), Chile (1), China (3), Ethiopia (1), Indonesia (1), Iraq (1), Mexico (1), Nigeria (1), Philippines (1), South Korea (1), Spain (1), Sri Lanka (5), Sudan (1), Syria (1), Tanzania (1), Timor-Leste (1), Turkey (6), Uganda (2), Vietnam (1)
Forced prolonged standing	24	14	7	1050	0.046	Bhutan (1), Bosnia and Herzegovina (1), China (1), Iraq (2), Pakistan (1), Palestinian Territory (1), Romania (1), Saudi Arabia (1), Somalia (1), Spain (2), Syria (1), Turkey (3), Uganda (1), Vietnam (1)

© 2023 Milewski A et al. *JAMA Network Open.*

Torture method	No. of studies	No. of countries	No. of UN subregions	No. of individuals	Average frequency	Countries where torture was reported to occur (number of studies for each country)
Stretching extremities	7	3	3	117	0.009	Ethiopia (1), India (3), Turkey (1)
Other physical torture	60	40	15	3334	0.143	Afghanistan (1), Algeria (1), Bosnia and Herzegovina (1), Cambodia (1), Cameroon (1), Chile (1), China (1), Congo DRC (1), Croatia (1), Côte d'Ivoire (1), Gambia (1), Germany (1), Greece (2), Guinea (1), India (4), Iran (4), Iraq (1), Israel (1), Lebanon (2), Liberia (1), Mali (1), Mauritania (1), Nepal (1), Nigeria (2), North Korea (1), Pakistan (1), Peru (1), Romania (1), Senegal (1), Serbia (1), Sierra Leone (1), Somalia (1), South Africa (1), Spain (3), Sri Lanka (2), Sudan (1), Togo (2), Turkey (3), Uganda (1), Vietnam (1)
Denailing	27	12	6	723	0.012	Bangladesh (1), Bhutan (1), Chile (1), China (1), Ethiopia (1), Iraq (1), Nepal (1), South Korea (1), Sri Lanka (4), Tanzania (1), Turkey (2), Vietnam (1)
Removal of teeth	13	4	3	93	0.008	Algeria (1), Ethiopia (1), Iran (1), Nepal (1)
Stoning	1	0	0	1	0.001	Unclear
Pharmacological torture	13	6	5	115	0.002	Chile (1), Ethiopia (1), Iraq (1), Peru (1), South Korea (1), Vietnam (1)
Chemicals or irritants	16	9	4	221	0.017	Bhutan (1), Chile (1), Côte d'Ivoire (1), India (1), Myanmar (1), Pakistan (1), Philippines (1), Sri Lanka (3), Thailand (1)
Exposure to extreme hot or cold	39	17	10	1563	0.057	Bhutan (1), Bosnia and Herzegovina (1), Chile (1), China (3), Honduras (1), India (1), Iraq (2), Israel (1), Palestinian Territory (1), Philippines (1), Romania (1), South Korea (1), Sri Lanka (1), Thailand (1), Turkey (2), United States/Guantánamo Bay (1), Vietnam (1)
Exposure to bright light	16	10	7	466	0.021	Cameroon (1), Chile (1), China (1), Congo DRC (1), Ethiopia (1), Ghana (1), Israel (1), South Africa (1), Sudan (1), Turkey (2)
Loud noises	9	5	3	680	0.014	Ethiopia (1), Iraq (2), Israel (1), Thailand (1), Turkey (2)
Deprivation of light	5	3	3	257	0.005	Ethiopia (1), Iran (1), Romania (1)
Other sensory torture	19	8	7	1619	0.021	Bhutan (1), Ethiopia (1), Mexico (1), South Korea (1), Sri Lanka (1), Sudan (1), Thailand (1), Turkey (2)
Rape	61	24	10	1754	0.083	Bosnia and Herzegovina (2), Cambodia (1), China (2), Congo DRC (1), Croatia (2), Ethiopia (1), Guatemala (1), Indonesia (1), Iraq (3), Israel (1), Mexico (1), Nepal (1),

© 2023 Milewski A et al. *JAMA Network Open*.

Torture method	No. of studies	No. of countries	No. of UN subregions	No. of individuals	Average frequency	Countries where torture was reported to occur (number of studies for each country)
						Nigeria (1), Palestinian Territory (1), Philippines (1), Saudi Arabia (1), Sierra Leone (1), Spain (1), Sri Lanka (5), Sudan (1), Syria (1), Tanzania (1), Turkey (3), Uganda (3)
Sexual assault or abuse	76	26	11	1383	0.093	Bangladesh (1), Chile (2), China (3), Congo DRC (1), Ethiopia (1), Greece (1), Iran (1), Iraq (2), Israel (1), Lebanon (2), Myanmar (1), Nepal (1), Nigeria (1), Palestinian Territory (1), Philippines (1), Saudi Arabia (1), Serbia (1), Sierra Leone (1), Spain (1), Sri Lanka (5), Sudan (1), Tanzania (1), Thailand (1), Turkey (4), Uganda (1), United States/Guantánamo Bay (1)
Sexual enslavement	2	1	1	3	0.002	Uganda (1)
Genital trauma	24	10	7	359	0.025	Ethiopia (1), Greece (2), Iraq (2), Israel (1), Mexico (1), Palestinian Territory (1), Spain (1), Sri Lanka (2), Sudan (1), Vietnam (1)
FGM/C	9	12	4	145	0.013	Burkina Faso (1), Cameroon (1), Central African Republic (1), Chad (1), Congo DRC (1), Côte d'Ivoire (1), Guinea (1), Mali (1), Nigeria (1), Sudan (1), Togo (1), Turkey (1)
Other sexual torture	44	15	9	1247	0.064	Cameroon (1), China (1), Egypt (1), Ethiopia (1), India (4), Indonesia (1), Israel (1), Mexico (1), Pakistan (1), Philippines (1), South Korea (1), Spain (2), Sri Lanka (2), Turkey (1), Uganda (1)
Sham executions	48	18	11	836	0.067	Chile (1), China (1), Congo DRC (1), Ethiopia (1), Greece (2), Iran (1), Iraq (1), Myanmar (1), Peru (1), Romania (1), Saudi Arabia (1), Somalia (1), South Korea (1), Spain (3), Sri Lanka (1), Turkey (4), United States/Guantánamo Bay (1), Vietnam (2)
Stripped naked	51	23	11	67210	0.08	Bhutan (1), Chile (2), China (4), Ethiopia (1), Honduras (1), India (1), Indonesia (1), Iraq (3), Israel (2), Lebanon (1), Mexico (2), Nigeria (1), Peru (1), Philippines (1), South Korea (1), Spain (4), Sri Lanka (3), Sudan (1), Syria (1), Tanzania (1), Thailand (1), Turkey (2), United States/Guantánamo Bay (1)
Verbal abuse or threats	86	41	14	78223	0.188	Afghanistan (1), Algeria (1), Bangladesh (1), Bhutan (1), Bosnia and Herzegovina (1), Burkina Faso (1), Cameroon (1), Chile (2), China (3), Congo DRC (1), Croatia (1), Egypt (1), Eritrea (1), Ethiopia (1), Germany (1), Honduras (1), India (2), Iran (2), Iraq (5), Israel (2), Lebanon (1), Libya (1), Malawi (1), Mexico (2), Nepal (1), Nigeria (1), North Korea (1), Palestinian Territory (1), Peru (1), Philippines (1), Romania (1), Serbia (1), Sierra Leone (1), South Korea (1), Spain (1), Sri Lanka (4), Sudan (1), Tanzania (1), Turkey (2), Uganda (1), United States/Guantánamo Bay (1)
Fluctuating interrogator attitude	6	4	4	299	0.008	Ethiopia (1), South Korea (1), Spain (1), Turkey (1)

© 2023 Milewski A et al. *JAMA Network Open.*

Torture method	No. of studies	No. of countries	No. of UN subregions	No. of individuals	Average frequency	Countries where torture was reported to occur (number of studies for each country)
Witnessing torture	84	29	13	5143	0.166	Algeria (1), Bosnia and Herzegovina (1), Cameroon (1), Chile (1), China (1), Congo DRC (1), El Salvador (1), Ethiopia (1), Germany (1), Guatemala (1), Iran (1), Iraq (3), Israel (1), Lebanon (1), Libya (1), Mexico (1), North Korea (1), Philippines (1), Romania (1), Serbia (1), Sierra Leone (1), South Korea (1), Spain (2), Sri Lanka (2), Syria (2), Tanzania (1), Thailand (1), Turkey (3), Vietnam (2)
Other psychological torture	132	48	15	12133	0.316	Albania (1), Argentina (1), Bangladesh (2), Bhutan (2), Bolivia (1), Bosnia and Herzegovina (3), Cambodia (1), Chile (4), China (2), Croatia (1), Côte d'Ivoire (1), El Salvador (1), Ethiopia (1), Germany (2), Ghana (1), Greece (2), Guatemala (1), Honduras (1), India (3), Indonesia (1), Iran (3), Iraq (8), Japan (1), Kyrgyzstan (1), Lebanon (3), Mexico (2), Nepal (1), Nigeria (2), North Korea (1), Pakistan (1), Palestinian Territory (1), Peru (2), Philippines (1), Romania (1), Saudi Arabia (1), Serbia (1), Somalia (1), South Korea (1), Spain (5), Sri Lanka (9), Sudan (1), Syria (2), Tanzania (1), Thailand (1), Turkey (7), Uruguay (1), United States/Guantánamo Bay (1), Vietnam (1)
Isolation or solitary confinement	71	29	13	3208	0.142	Afghanistan (1), Bhutan (2), Bosnia and Herzegovina (1), Chile (1), China (3), Croatia (1), Ethiopia (1), Germany (1), Ghana (1), Greece (2), Honduras (1), Iran (2), Iraq (3), Israel (1), Lebanon (2), Mexico (1), Nepal (1), North Korea (1), Palestinian Territory (1), Romania (1), Saudi Arabia (1), South Africa (1), South Korea (1), Spain (3), Sri Lanka (2), Thailand (1), Turkey (4), United States/Guantánamo Bay (1), Vietnam (2)
Blindfolding	49	23	11	53303	0.104	Bangladesh (1), Cambodia (1), Chile (2), El Salvador (1), Ethiopia (1), Honduras (1), India (2), Iraq (2), Israel (1), Lebanon (1), Mexico (2), Nigeria (1), Philippines (1), Romania (1), South Korea (1), Spain (2), Sri Lanka (4), Syria (1), Tanzania (1), Thailand (1), Turkey (5), United States/Guantánamo Bay (1), Vietnam (1)
Prevention of hygiene or exposure to infested surroundings	45	20	10	2236	0.085	Bhutan (2), Chile (1), China (1), Congo DRC (1), Ethiopia (1), Honduras (1), India (1), Iran (1), Iraq (4), Nepal (1), North Korea (1), Peru (1), Philippines (1), Spain (1), Sri Lanka (2), Sudan (1), Tanzania (1), Thailand (1), Turkey (4), Vietnam (1)
Sleep deprivation	35	16	9	2079	0.062	Bhutan (2), Chile (2), China (2), Ethiopia (1), Greece (2), Iraq (3), Israel (1), Peru (1), Philippines (1), Romania (1), Saudi Arabia (1), South Korea (1), Spain (3), Turkey (3), United States/Guantánamo Bay (1), Vietnam (1)

© 2023 Milewski A et al. *JAMA Network Open.*

eTable 9. Countries in Which Individuals Who Experienced Torture Were Encountered for Study

Country	N	Country	N	Country	N
Albania	1	Ireland	3	Romania	1
Australia	5	Israel	2	South Africa	5
Austria	5	Italy	5	South Korea	1
Bangladesh	2	Jordan	3	Spain	10
Bosnia and Herzegovina	1	Kyrgyzstan	1	Sri Lanka	7
Cambodia	1	Lebanon	3	Sudan	1
Canada	10	Malaysia	1	Sweden	6
Chile	2	Mexico	1	Switzerland	3
Croatia	6	Nepal	5	Syria	1
Denmark	32	Netherlands	3	Tanzania	1
Egypt	2	North Macedonia	1	Thailand	1
France	6	Norway	5	Timor-Leste	1
Germany	7	Pakistan	2	Tunisia	1
Ghana	1	Palestine	5	Turkey	10
Greece	5	Peru	2	Uganda	3
Honduras	2	Philippines	1	United Kingdom	21
India	12	Portugal	1	United States*	64
Iraq	3				

N = number of studies
*Includes 1 study wherein the individuals were encountered in Guantanamo Bay Naval Base

© 2023 Milewski A et al. *JAMA Network Open.*

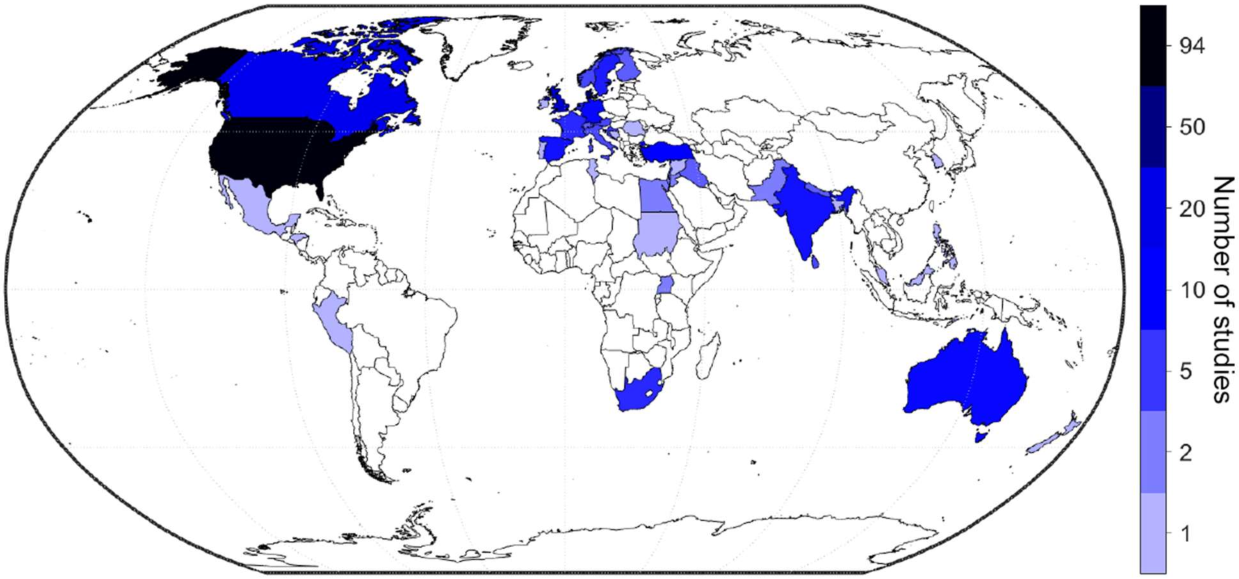

eFigure 5. Countries Hosting Researchers
Countries hosting the investigators who performed the research. Darker shades of blue indicate that a greater number of studies were performed in the country. e**Table 8** lists the countries that hosted the researchers.

© 2023 Milewski A et al. *JAMA Network Open.*

eTable 10. Countries Hosting Researchers

Country	N	Country	N	Country	N
Australia	9	Israel	2	Romania	1
Austria	4	Italy	4	South Africa	6
Bangladesh	1	Jordan	2	South Korea	1
Bosnia and Herzegovina	1	Lebanon	4	Spain	8
Canada	18	Malaysia	1	Sri Lanka	4
Croatia	5	Mexico	1	Sudan	1
Denmark	51	Nepal	3	Sweden	7
Egypt	2	Netherlands	6	Switzerland	3
Finland	3	New Zealand	1	Syria	1
France	6	Norway	4	Timor-Leste	1
Germany	9	Pakistan	2	Tunisia	1
Honduras	1	Palestine	3	Turkey	10
India	8	Peru	1	Uganda	2
Iraq	3	Philippines	1	United Kingdom	30
Ireland	1	Portugal	1	United States	94

N = number of studies
A single study may have multiple sponsors. Additionally, one study was partly sponsored by Taiwan.
Researchers were hosted by a single country for 218 studies, by two countries for 41 studies, and by three or more countries for seven studies.

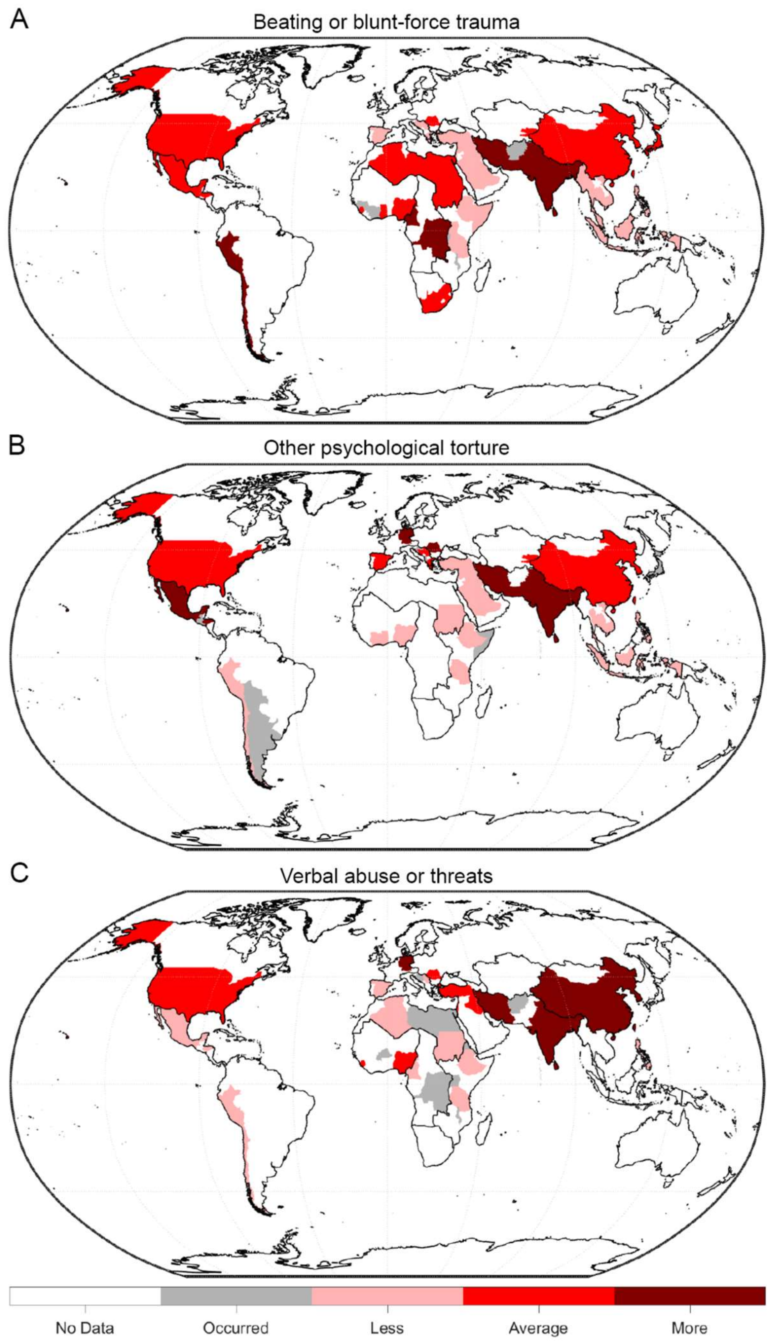

eFigure 6. Regional Variation for the Top 3 Torture Methods
Shaded areas indicate countries in which beating or blunt-force trauma (A); other psychological torture (B); or verbal abuse or threats (C) occurred. Light-red indicates that the frequency of the torture method was statistically less than average for that UN subregion compared to all other subregions combined, red indicates that the frequency of the torture method was not statistically different from average for that UN subregion, and dark-red indicates that the frequency of the torture method was statistically greater than average. Gray indicates that the torture method was reported for the area, but a frequency could not be determined. Fisher's exact test was used to evaluate the difference in the frequency of a torture method between an individual UN subregion and all other subregions combined. Only those individuals who could clearly be ascribed to a specific UN subregion were included in the analysis. For all of the statistically significant results shown, p < 1.5e-6. To avoid introducing a major bias from a single, large study, this analysis excludes the study with the greatest number of individuals (Magaloni 2020).

© 2023 Milewski A et al. *JAMA Network Open.*

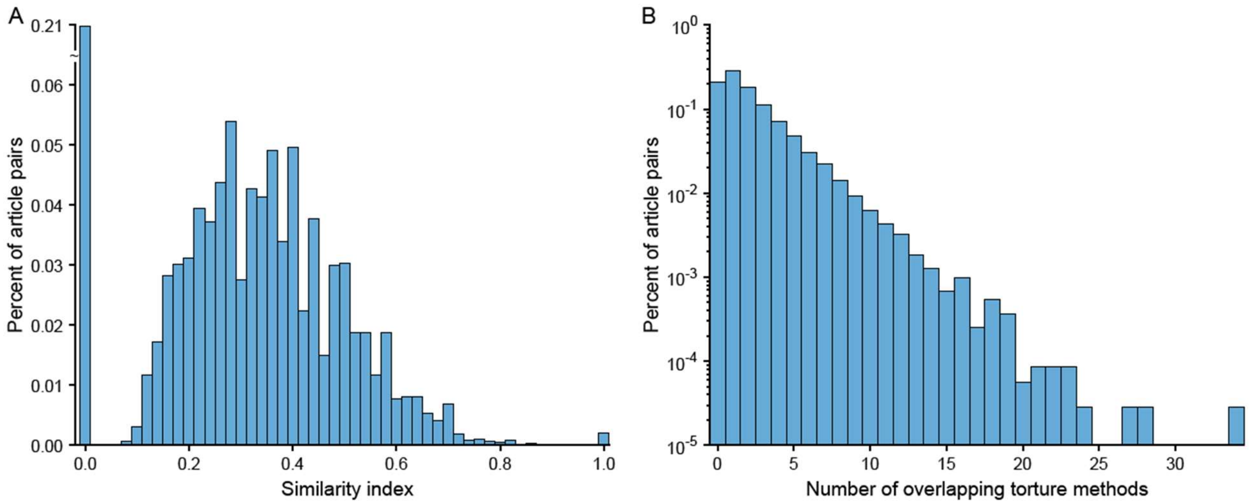

eFigure 7. Distributions for the Similarity Index and the Number of Overlapping Torture Methods
(A) A bar graph displays the distribution of the similarity indices for all pairs of articles. Although 0.0 was the most frequent similarity index, some degree of overlapping torture methods occurred for nearly 80% of pairs of articles. (B) A bar graph portrays the distribution for the number of torture methods reported by both articles in every possible pairing of articles. There were no methods in common for 20.1% of article pairs, one method in common for 28.4% of articles pair, two methods in common for 18.0% of article pairs, three methods in common for 11.3% of article pairs, four methods in common for 7.1% of article pairs, five methods in common for 4.8% of article pairs, and more than five methods in common for 10.3% of articles. To allow for better visualization of the entire distribution, the ordinate axis is shown on a logarithmic scale.

© 2023 Milewski A et al. *JAMA Network Open.*

eTable 11. Assessment of Risk of Bias Using the Downs and Black Checklist

Study ID	Down & Black Sum	Quality Level
Aalund 1990	13	Poor
Abildgaard 1984	16	Fair
Adinkrah 2011	9	Poor
Agger 1990	10	Poor
Aksaray 2000	8	Poor
Alayarian 2009	10	Poor
Allden 1998	6	Poor
Allodi 1982	11	Poor
Allodi 1990	19	Fair
Alpak 2015	15	Fair
Alqassab 2021	11	Poor
Altun 2003	8	Poor
AmoneP'Olak 2009	20	Good
Amris 2007	10	Poor
Amris 2009	16	Fair
Aon 2018	21	Good
Arge 2014	10	Poor
Aron 1991	10	Poor
Asgary 2006	15	Fair
de Zoysa 2007	11	Poor
Asgary 2013	15	Fair
Asirdizer 2004	12	Poor
Badiee 2015	4	Poor
Barber 2011	9	Poor
Basoglu 1992	11	Poor
Basoglu 1997	24	Good
Basoglu 2004	13	Poor
Berger 1980	10	Poor
Berman 2006	10	Poor
Bichescu 2005	24	Good
Bloom 1995	10	Poor
Boersma 2003	10	Poor
Boos 2000	12	Poor
Bork 1997	9	Poor
Bouwer 1998	18	Fair
Bradley 2006	16	Fair
BroddaJansen 2020	14	Poor
Busch 2015	19	Fair
Byard 2012	9	Poor
Callaghan 1993	10	Poor
Carlsson 2006	23	Good

© 2023 Milewski A et al. *JAMA Network Open.*

Study ID	Down & Black Sum	Quality Level
Cathcart 1979	10	Poor
Charlier 2018	11	Poor
Chaudhry 2008	14	Poor
Choi 2017	22	Good
Chu 2013	22	Good
Clement 2017	19	Fair
Cohen 2017	21	Good
Cooper 2011	8	Poor
Crescenzi 2002	23	Good
Crosby 2010	10	Poor
Dalgaard 2021	19	Fair
DandeniyaArachchi 2019	14	Poor
Danielsen 1981	10	Poor
Danneskiold-Samsoe 2006	13	Poor
Daugaard 1983	22	Good
deFouchier 2012	15	Fair
deJong 2008	23	Good
Deol 2018	20	Good
Deps 2021	17	Fair
Deps 2021	16	Fair
Dibaj 2017	17	Fair
Dickson-Lowe 2021	13	Poor
Dolma 2006	14	Poor
Domovitch 1984	14	Poor
El Sarraj 1996	20	Good
East 2018	11	Poor
Edston 2007	15	Fair
Einolf 2018	12	Poor
Engstrom 2004	10	Poor
Fahy 1988	10	Poor
Fernandes 2019	12	Poor
Fernandez 2001	11	Poor
Ferrada-Noli 1998	18	Fair
Fetherston 2020	11	Poor
Fidaner 1991	18	Fair
Fischman 1990	11	Poor
Fornazzari 1990	18	Fair
Forrest 1995	10	Poor
Gavagan 1997	10	Poor
Ghaddar 2016	22	Good
Ghaleb 2014	21	Good
Gniadecka 1995	7	Poor

© 2023 Milewski A et al. *JAMA Network Open.*

Study ID	Down & Black Sum	Quality Level
Gonsalves 1990	15	Fair
Gorst-Unsworth 1993	13	Poor
Gorst-Unsworth 1998	21	Good
Gray 2001	25	Good
Green 2007	9	Poor
Gregurek 2001	10	Poor
Grodin 2008	10	Poor
Grossman 1996	20	Good
Gulden 2010	20	Good
Guzel 2015	10	Poor
Haar 2019	16	Fair
Halvorsen 2010	19	Fair
Harris 2019	10	Poor
Hartmann 2009	19	Fair
Hexom 2012	15	Fair
Highfield 2012	16	Fair
Holtz 1998	17	Fair
Hondius 2000	16	Fair
Hooberman 2010	16	Fair
Hougen 1988	16	Fair
Hougen 1988	16	Fair
Hughes 2012	11	Poor
Hunt 2008	16	Fair
Iacopino 2001	17	Fair
Iacopino 2011	11	Poor
Ibrahim 2017	17	Fair
Ilenia 2021	15	Fair
Jayawickreme 2019	12	Poor
Jayawickreme 2020	21	Good
Jensen 2013	10	Poor
Jorgensen 2015	23	Good
Kagee 2004	10	Poor
Kanninen 2000	17	Fair
Kastrup 1986	13	Poor
Kaur 2020	22	Good
Keatley 2015	26	Excellent
Keller 1998	10	Poor
Keller 2002	8	Poor
Keller 2006	10	Poor
Keller 2006	21	Good
Keller 2014	10	Poor
Keten 2020	9	Poor

© 2023 Milewski A et al. *JAMA Network Open.*

Study ID	Down & Black Sum	Quality Level
Kienzler 2019	14	Poor
Kinyanda 2010	14	Poor
Kinzie 1987	10	Poor
Kinzie 2012	19	Fair
Kizilhan 2018	8	Poor
Knipscheer 2015	20	Good
Kozaric-Kovacic 1995	11	Poor
Kucukalic 2003	16	Fair
Larsen 1987	9	Poor
Lawson 1999	13	Poor
Leaman 2012	18	Fair
Lerner 2016	20	Good
Leth 2005	14	Poor
Ley 2018	19	Fair
Lie 2002	10	Poor
Loncar 2006	20	Good
Loncar 2010	12	Poor
Longstreth 2021	11	Poor
Loutan 1999	22	Good
Lunde 1990	20	Good
Lykke 2002	13	Poor
Magaloni 2020	22	Good
Magli 2019	10	Poor
Malik 1993	13	Poor
Malik 1995	13	Poor
Mannan 2011	10	Poor
Martell 2020	11	Poor
Masmas 2008	19	Fair
Matos 2021	14	Poor
McColl 2010	18	Fair
McKenzie 2017	11	Poor
MemberCentersoftheNationalConsortiumofTortureTreatmentPrograms(NCTTP) 2015	6	Poor
Miller 1989	15	Fair
Mirzaei 1998	16	Fair
Moisander 2003	17	Fair
Mollica 1998	23	Good
Mollica 2007	22	Good
Mollica 2014	24	Good
Montgomery 1998	10	Poor
Moreno 2000	10	Poor

Study ID	Down & Black Sum	Quality Level
Moreno 2003	14	Poor
Moreno 2006	18	Fair
Moreno 2008	13	Poor
Moreno 2015	15	Fair
Moreno 2020	13	Poor
Morentin 1995	19	Fair
Morentin 1997	13	Poor
Morentin 2008	18	Fair
Munczek 1998	10	Poor
Musisi 2000	23	Good
Neufeld 2021	15	Fair
Nguyen 2019	12	Poor
Nickerson 2016	20	Good
Nieves-Grafals 2001	11	Poor
Nordin 2019	18	Fair
Norredam 2005	10	Poor
Nou 2015	14	Poor
Olsen 2006	20	Good
Olsen 2006	18	Fair
Olsen 2007	20	Good
Owens 2021	12	Poor
Ozkalipci 2013	17	Fair
Peel 1996	12	Poor
Peel 2000	12	Poor
Peel 2003	9	Poor
Peltzer 1999	10	Poor
Perez-Sales 2010	10	Poor
Perez-Sales 2016	13	Poor
Petersen 1985	16	Fair
Petersen 1985	21	Good
Petersen 1994	9	Poor
Petersen 1995	9	Poor
Petersen 2000	19	Fair
Petersen 2019	17	Fair
Piwowarczyk 2017	18	Fair
Pohlman 2017	10	Poor
Polat 2010	10	Poor
Pollanen 2002	9	Poor
Pollanen 2003	9	Poor
Pollanen 2016	9	Poor
Priebe 1997	19	Fair
Prip 2008	17	Fair

© 2023 Milewski A et al. *JAMA Network Open.*

Study ID	Down & Black Sum	Quality Level
Prip 2011	21	Good
Prip 2012	22	Good
Punamaki 1988	14	Poor
Ramsay 1993	17	Fair
RanilSanjeewa 2017	11	Poor
Rashid 2012	13	Poor
Rasmussen 1980	14	Poor
Rasmussen 1990	20	Good
Rasmussen 2007	21	Good
Rasmussen 2007	15	Fair
Rasmussen 2011	22	Good
Ray 2006	17	Fair
Reid 1988	6	Poor
Reid 1990	12	Poor
Rivera-Holguin 2019	14	Poor
Rodolico 2020	13	Poor
RoufKhawaja 2020	16	Fair
Montgomery 2004	19	Fair
Saab 2003	13	Poor
Saadi 2021	10	Poor
Perera 2007	13	Poor
Sachs 2008	22	Good
Sanders 2009	12	Poor
Sangmo 2020	18	Fair
Savnik 2000	16	Fair
Savy 2008	10	Poor
Schnyder 2015	14	Poor
Sen 2018	6	Poor
Singh 2012	13	Poor
SmithFawzi 1997	10	Poor
Somnier 1986	11	Poor
Song 2015	23	Good
Sutker 1990	13	Poor
Tamblyn 2011	18	Fair
Thomsen 2000	12	Poor
Torp-Pedersen 2009	18	Fair
Tran 2020	18	Fair
Tsai 2012	15	Fair
Unuvar 2014	10	Poor
Van Ommeren 1998	26	Excellent
Van Ommeren 2001	19	Fair
Van Velsen 1996	21	Good

© 2023 Milewski A et al. *JAMA Network Open.*

Study ID	Down & Black Sum	Quality Level
VillerHansen 2017	16	Fair
Vohra 2019	10	Poor
Vrca 1996	19	Fair
Wang 2009	21	Good
Weinstein 1996	10	Poor
Weisaeth 1989	15	Fair
Weishut 2015	14	Poor
Wenzel 2000	12	Poor
Westermeyer 2011	22	Good
Wikholm 2020	20	Good
Williams 2010	19	Fair
Wilson 2013	17	Fair
Wolf 1947	10	Poor
Womersley 2018	13	Poor
Young 2008	10	Poor
Zandieh 2016	19	Fair
Zech 2017	14	Poor

© 2023 Milewski A et al. *JAMA Network Open.*

eTable 12. Number of Articles Satisfying Each Downs and Black Checklist Item

Downs & Black Checklist Item	No. Articles
**REPORTING**	
Is the objective of the study clear?	262
Are the main outcomes clearly described in the Introduction or Methods?	261
Are characteristics of the patients included in the study clearly described?	252
Are the interventions clearly described?	266
Are the distributions of principal confounders in each group of subjects clearly described?	118
Are the main findings of the study clearly described?	261
Does the study estimate random variability in data for main outcomes?	26
Have all the important adverse events consequential to the intervention been reported?	240
Have characteristics of patients lost to follow-up been described?	36
Have actual probability values been reported for the main outcomes except probability < 0.001?	94
Is the source of funding clearly stated?	96
Were subjects who were asked to participate in the study representative of the entire population recruited?	162
Were those subjects who were prepared to participate representative of the recruited population?	162
Were staff, places, and facilities where patients were treated representative of treatment most received?	260
**EXTERNAL VALIDITY**	
Were subjects who were asked to participate in the study representative of the entire population recruited?	79
Were those subjects who were prepared to participate representative of the recruited population?	79
Were staff, places, and facilities where patients were treated representative of treatment most received?	259
**INTERNAL VALIDITY**	
Was an attempt made to blind study subjects to the intervention?	3
Was an attempt made to blind those measuring the main outcomes?	5
If any of the results of the study were based on data dredging was this made clear?	0
Was the time period between intervention and outcome the same for intervention and control groups or adjusted for?	36
Were the statistical tests used to assess main outcomes appropriate?	117
Was compliance with the interventions reliable?	263
Were main outcome measures used accurate? (valid and reliable)	260
**INTERNAL VALIDITY-CONFOUNDING (SELECTION BIAS)**	
Were patients in different intervention groups recruited from the same population?	56
Were study subjects in different intervention groups recruited over the same period of time?	57
Were study subjects randomized to intervention groups?	11
Was the randomized intervention assignment concealed from patients and staff until recruitment was complete?	2
Was there adequate adjustment for confounding in the analyses from which main findings were drawn?	68
Were losses of patients to follow-up taken into account?	8
**POWER**	
Was the study sufficiently powered to detect clinically important effects where probability value for a difference due to chance is < 5%?	111

© 2023 Milewski A et al. *JAMA Network Open.*
