# Supplementary material for: Reported Methods, Distributions, and Frequencies of Torture Globally: A Systematic Review and Meta-Analysis
Source: JAMA Netw Open. 2023 Oct 3;6(10):e2336629. doi: 10.1001/jamanetworkopen.2023.36629 (PMC10548313; doi:10.1001/jamanetworkopen.2023.36629)
Supplement: Supplement 2. — Data Sharing Statement [file jamanetwopen-e2336629-s002.pdf]

Data Sharing Statement

Milewski. Reported Methods, Distributions, and Frequencies of Torture Globally. *JAMA Netw Open*. Published October 03, 2023. doi:10.1001/jamanetworkopen.2023.36629

Data
Data available: Yes
Data types: Data (not involving human participants), Data dictionary
How to access data: anm2119@med.cornell.edu
When available: With publication

Supporting Documents
Document types: Statistical/analytic code
How to access documents: anm2119@med.cornell.edu
When available: With publication

Additional Information
Who can access the data: Anyone requesting the data
Types of analyses: For any purpose
Mechanisms of data availability: With investigator support
